# Non-invasive prenatal diagnosis of single gene disorders with enhanced relative haplotype dosage analysis for diagnostic implementation

**DOI:** 10.1371/journal.pone.0280976

**Published:** 2023-04-24

**Authors:** Mathilde Pacault, Camille Verebi, Magali Champion, Lucie Orhant, Alexandre Perrier, Emmanuelle Girodon, France Leturcq, Dominique Vidaud, Claude Férec, Thierry Bienvenu, Romain Daveau, Juliette Nectoux

**Affiliations:** 1 Laboratoire de Génétique Moléculaire et Histocompatibilité, Brest, France; 2 Service de Médecine Génomique des maladies de système et d’organe, APHP.Centre - Université Paris Cité, Hôpital Cochin, Paris, France; 3 Université de Paris, CNRS, MAP5 UMR8145, Paris, France; 4 MOABI, Plateforme bio-informatique AP-HP, Département I&D, DSI, Paris, France; Universita degli Studi di Roma Tor Vergata, ITALY

## Abstract

Non-invasive prenatal diagnosis of single-gene disorders (SGD-NIPD) has been widely accepted, but is mostly limited to the exclusion of either paternal or *de novo* mutations. Indeed, it is still difficult to infer the inheritance of the maternal allele from cell-free DNA (cfDNA) analysis. Based on the study of maternal haplotype imbalance in cfDNA, relative haplotype dosage (RHDO) was developed to address this challenge. Although RHDO has been shown to be reliable, robust control of statistical error and explicit delineation of critical parameters for assessing the quality of the analysis have not been fully addressed. We present here a universal and adaptable enhanced-RHDO (eRHDO) procedure through an automated bioinformatics pipeline with a didactic visualization of the results, aiming to be applied for any SGD-NIPD in routine care. A training cohort of 43 families carrying *CFTR*, *NF1*, *DMD*, *or F8* mutations allowed the characterization and optimal setting of several adjustable data variables, such as minimum sequencing depth, type 1 and type 2 statistical errors, as well as the quality assessment of intermediate steps and final results by block score and concordance score. Validation was successfully performed on a test cohort of 56 pregnancies. Finally, computer simulations were used to estimate the effect of fetal-fraction, sequencing depth and number of informative SNPs on the quality of results. Our workflow proved to be robust, as we obtained conclusive and correctly inferred fetal genotypes in 94.9% of cases, with no false-negative or false-positive results. By standardizing data generation and analysis, we fully describe a turnkey protocol for laboratories wishing to offer eRHDO-based non-invasive prenatal diagnosis for single-gene disorders as an alternative to conventional prenatal diagnosis.

## Introduction

The identification of fetal DNA in maternal blood by Dennis Lo in 1997 has dramatically changed the landscape of prenatal diagnosis (PND) [1]. The most significant impact to date has probably been the widespread use of cell-free DNA (cfDNA) testing for common fetal aneuploidies and, more recently, subchromosomal abnormalities [2–5], which has been widely accepted by clinicians and pregnant women [6], dramatically reducing the invasive sampling rate for this indication over a few years [7, 8]. Detection of fetal-specific sequences that are absent or different from the maternal genome is also routinely performed either for fetal sex determination [9, 10], fetal RhD genotyping [11, 12] or exclusion of paternally-inherited or *de novo* mutations in fetuses at risk for single-gene disorders (SGD) [13–15]. However, determination of fetal status with respect to maternal pathogenic variants remains more challenging, because haploidentical maternal and fetal sequences cannot be easily distinguished.

While the detection of paternally inherited or *de novo* variants is based on a qualitative presence/absence approach, most workflows described to date for maternal inheritance determination rely on the fine quantification of each maternal haplotype, called relative haplotype dosage analysis (RHDO), originally described by Lo *et al*. [16]. In this approach, based on massively parallel sequencing (MPS), parental, proband and cfDNA are sequenced in parallel at multiple polymorphic positions along the locus of interest. The two haplotypes of each parent are phased by comparison with genotype of the proband. Paternal inheritance is then determined based on the qualitative detection of polymorphisms where the two paternal alleles can be distinguished, i.e. where the father is heterozygous and the mother is homozygous. In contrast, maternal inheritance is based on the calculation of an allelic ratio at positions where the two maternal alleles can be differentiated, i.e. where the mother is heterozygous and the father is homozygous. The detection of a haplotype imbalance would reflect the fetal contribution, from which we can deduce the maternal haplotype that was transmitted to the fetus, healthy or at-risk. By relying on indirect diagnosis, RHDO theoretically allows diagnosis for any family, regardless of the inheritance pattern or the type of molecular abnormality and has been successfully applied to several SGDs [17–21]. However, to our knowledge, clinical implementation in public health service laboratories around the world remains sparse, probably because quality controls and decision thresholds remain complex to define [22–24].

In this report, we describe our RHDO-based NIPD workflow, which we call *enhanced*-RHDO (eRHDO), where we explain in detail our bioinformatics and statistical analyses with precise risk control and present a straightforward and comprehensive result visualization. Using computer simulations, we discuss the impact of the different biological and analytical parameters on the quality of the result, and propose thresholds and objective quality scores for easy implementation in a diagnostic laboratory.

## Materials and methods

### Participants and sample processing

Pregnant women known to be carriers of cystic fibrosis (*CFTR* gene), neurofibromatosis type 1 (*NF1* gene), Duchenne/Becker muscular dystrophy (*DMD* gene) or hemophilia (*F8* and *F9* genes) were recruited nationwide through the DANNI and NID studies, which were ethically approved by the French "Comité Consultatif sur le Traitement de l’Information en matière de Recherche dans le domaine de la Santé” (ref. 13.386) and the "Comité de Protection des Personnes” (ref. 2014-janvier-13465 and 29BRC18.0055). The French law regulating PND and genetic testing requires approval of the indication for referral by the local ethics committee and written informed consent from both parents. All data were fully anonymized. We divided our cohort into two groups. The first group, called "training cohort", included families for whom only parental, fetal and cfDNA were available, i.e. parental haplotypes were determined using fetal information from the current pregnancy. Blood samples from women in this group were initially used to validate the efficiency, accuracy and multiplexing capacity of our method. When genomic DNA (gDNA) from a first child or close relative was available, pregnant women were included in the “test cohort”. For these patients, we used the proband’s gDNA to identify each parental haplotypes, allowing these families to be tested in a real-world setting.

**Fig 1:**
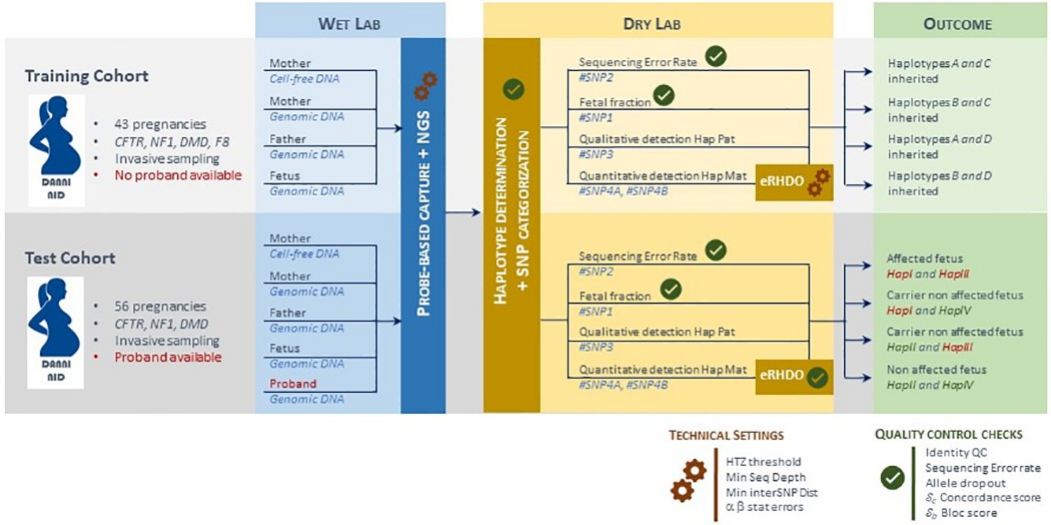
Workflow and processing steps for samples obtained from the training and test cohorts. The training cohort included couples at risk for SGD who were offered invasive prenatal testing and for whom parental haplotypes were determined using fetal genomic DNA from the current pregnancy. Test cohort included pregnant women at risk for SGD who were offered invasive prenatal testing and for whom a proband’s genomic DNA was available for haplotype reconstruction. The training cohort study allowed to evaluate and optimize the technical settings. The best combination of settings was then used to explore the test cohort. Finally, the diagnostic performance of our SGD-NIPD workflow with eRHDO was evaluated and compared to gold standard as defined by fetal status obtained by invasive sampling. Quality controls measures are described to minimize errors and to aid in technical validation and biological interpretation of the result.

The DNA samples required for each family included the cfDNA extracted from maternal plasma, maternal and paternal gDNA extracted from leukocytes, the proband gDNA if available and the fetal gDNA from invasive sampling to confirm the NIPD result (Fig 1). All samples from the same family were processed simultaneously and pooled prior to targeted capture enrichment and MPS. Details of DNA extraction and sample processing for MPS library preparations are provided in the supporting information. After targeted MPS, sequencing data from each DNA sample was used to perform eRHDO analysis and determine fetal inheritance at the locus of interest. The results were set as haplotype A, B, C or D for the training cohort, as the goal was to refine analysis thresholds and determine whether the fetal haplotype was correctly inferred, regardless of its risk for SGD. For the test cohort, we studied only the gene involved in familial SGD, and the results were set as "HapI" or "HapII" for maternal at-risk or non-at-risk haplotype; and "HapIII" or "HapIV" for paternal at-risk or non-at-risk haplotype, respectively.

### SGD-NIPD workflow: Processing steps and terminology

After sequencing, a family-specific pile-up was generated by counting the number of reads for each base, from the parental at-risk and non-at-risk haplotypes, at each SNP targeted in our capture panel. The two maternal haplotypes are HapI and HapII, where HapI is the maternal at-risk haplotype and HapII is the maternal non-at-risk haplotype; while the two paternal haplotypes are HapIII and HapIV, where HapIII is the paternal at-risk haplotype and HapIV is the paternal non-at-risk haplotype.

Each SNP of the pile-up was categorized based on parental inheritance, as originally described by Lo *et al*. [16]. Briefly, SNP1 are defined as those for which the father and mother are both homozygous for different alleles (mother AA and father BB). SNP2 are those where the father and mother are both homozygous for the same allele (mother AA and father AA). SNP3 are those for which the father is heterozygous and the mother is homozygous (mother AA and father AB). Finally, SNP4 are those for which the father is homozygous and the mother is heterozygous (mother AB and father AA). SNP4 can be subdivided into SNP4a and SNP4b. SNP4a are those for which the paternal allele is the same as the maternal allele from HapI, while SNP4b are those for which the paternal allele is the same as the maternal allele from HapII. The other genomic positions of the pileup are categorized as WARNING_1 or WARNING_2 (S1 and S2 Tables).

SNPs from each category were then analyzed in maternal plasma by SGD-NIPD analysis to simultaneously provide i/ sequencing error rate determination (SNP2 analysis), ii/ fetal fraction assessment (SNP1 analysis or uncategorized SNP analysis), iii/ qualitative paternal haplotype transmission determination (SNP3 analysis), and iv/ quantitative maternal haplotype transmission determination (SPRT-based eRHDO SNP4 analysis). After technical validation of the result, biological interpretation and medical conclusion can be performed.

**Fig 2:**
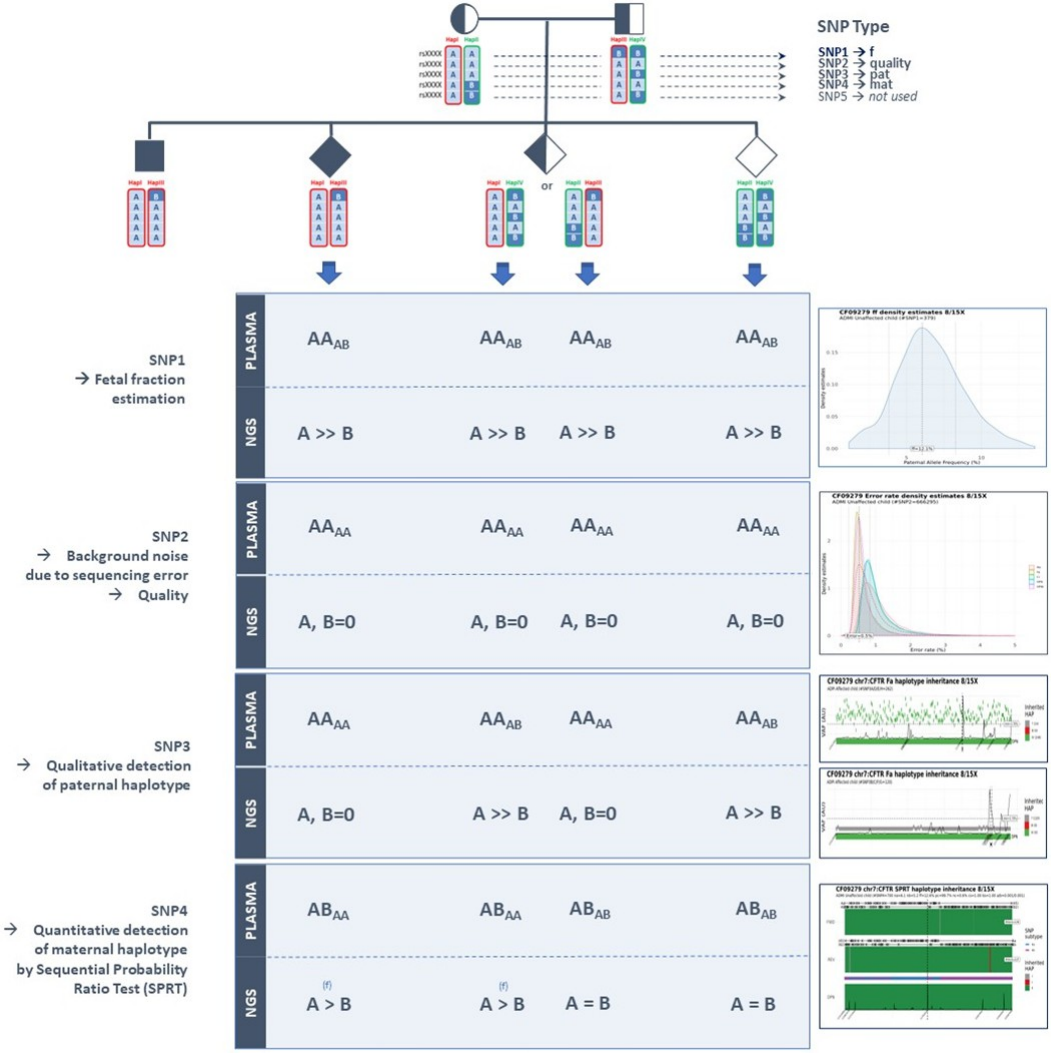
Noninvasive fetal genomic analysis from maternal plasma DNA. In the test cohort, the proband’s gDNA is used to identify each parental haplotypes. HapI is the maternal at-risk haplotype; HapII is the maternal non-at-risk haplotype, HapIII is the paternal at-risk haplotype and HapIV is the paternal non-at-risk haplotype. A family-specific pile-up was generated from cfDNA sequencing data, by counting the number of reads for each base. Each SNP of the pile-up can be categorized based on parental inheritance. SNP1 (mother AA and father BB) and SNP2 (mother AA and father AA) allow the basic parameters for maternal plasma DNA sequencing to be established, including fetal fraction and sequencing error rate estimations. SNP3 (mother AA and father AB) allow qualitative paternal haplotype transmission determination. SNP4 (mother AB and father AA) allow quantitative maternal haplotype transmission determination, thanks the tracking of fetal inheritance of a haplotype block close to the mutation carried by the mother. SNP5 were not analyzed in this study.

The SGD-NIPD workflow is shown in Fig 1 and the principles of noninvasive fetal genomic analysis from maternal plasma DNA are depicted in Fig 2.

### Data analysis

Using simulated cfDNA sequencing data and data from the training cohort, we defined adjustable data variables, namely consideration of thresholds used to discriminate a heterozygous position in gDNA and minimum sequencing depth for SNP calling in gDNA (NDP) and cfDNA (PDP). Minimum interSNP distance for eRHDO analysis, and Type I and Type II error risks associated with SPRT haplotype block delineation were defined as output settings. We then tested different combinations of input and output settings and objectively examined their impact on the quality scores in order to select the best combination of input and output settings for test cohort exploration. Finally, the diagnostic performance of our noninvasive test was determined from the test cohort analysis.

Details of the bioinformatics pipeline for sequencing data analysis are provided in S1 File. We generated a graphical visualization of the results, including preanalytical and analytical information, such as input data (inheritance mode, proband sex for X-linked disorders), fetal fraction, and quality parameters (position of the parental variant, total number of SNPs called for each analysis and distance between tested SNPs). For the eRHDO analysis, the repartition of SNP4a and SN4b along the locus, the mean number of SNPs per haplotype block, the number of haplotype blocks, the concordance between forward- and reverse-defined blocks (*pc*), and the proportion of positions where the SPRT remained non-conclusive (*nc*) are reported. SNPs were arbitrarily colored in green for the wild-type-linked haplotype, in red for the mutant-linked haplotype, and in gray for inconclusive results. This entire analysis pipeline was developed and refined using our training cohort. Its reliability and robustness, as well as the appropriateness of the chosen thresholds were tested on our test cohort. In this research study, all results were compared with the fetal genotype obtained on invasive sampling.

### Quality parameters for eRHDO analysis

We implemented quality control tags as well as two eRHDO-specific quality scores, which we named concordance score $S_{c}$ and block score $S_{b}$, with the aim of reflecting forward and reverse concordance, haplotype classification error rate as well as the ease of concluding in favor of the overrepresentation of one of the maternal haplotypes.

The concordance score *S*_*c*_ is defined as the difference between the concordance proportion between forward and reverse analyses *pc* and the proportion of SNPs that remained inconclusive *nc*. To strongly discriminate between misclassified haplotype blocks (low *pc*) and low-quality results (high *nc*), which would reflect a low quality result to be interpreted with caution, *S*_*c*_ is calculated as follows: *S*_*c*_ = −*log*_100_(1 − *pc*) − *nc*. A *S*_*c*_ closer to 1 reflects a more robust result.

The block score $S_{b}$ is obtained by normalizing the averaged number of SNPs per haplotype block by the total number of SNPs tested on the locus, expressed in a logarithmic scale for better discrimination: $S_{b}=-{log}_{100}(\frac{\bar{SNPs\;per\;block}}{\#SNPs})$. Shorter haplotype blocks mean that fewer positions were tested before reaching statistical significance. In other words, a block score close to 1 reflects a greater imbalance between the two maternal alleles, and therefore a higher confidence in the fetal haplotype being called.

### Statistical analysis

All statistical analyses were performed using R. To measure the effect of the settings on the quality of the results, we performed Wilcoxon tests and Friedman tests for variables with two (heterozygous position in gDNA) or more than two groups (minimum sequencing depth and minimum interSNP distance). In addition, we presented the associations using box plots.

The effect of all quality influencing factors (fetal fraction, number of SNPs, and sequencing depth) and their combinations on the quality of the results was quantified using Sobol sensitivity indices, ranging from 0 (minor effect) and 100 (major effect). P-values for ANOVA tests were also calculated to verify their significance. All p-values were adjusted for multiple comparisons using the Bonferroni correction.

## Results

### Patient information

The training cohort included 43 pregnancies, divided into 27 pregnancies at 25% risk for cystic fibrosis, 9 pregnancies at 50% risk for maternal transmission of neurofibromatosis type 1, and 3 and 4 pregnancies with a male fetus at 50% risk for Duchenne/Becker muscular dystrophy and hemophilia, respectively. Gestational age at sampling ranged from 6 to 29 weeks of gestation (mean, 12 weeks).

The test cohort included 56 pregnancies from 45 families, divided into 25 pregnancies at 25% risk of cystic fibrosis (paternal and maternal haplotypes transmission were determined noninvasively in an autosomal recessive pattern if both parents carried the same mutation, or in an autosomal paternal + maternal dominant pattern if parents carried different mutations), 11 pregnancies with a 50% risk of maternal transmission of neurofibromatosis type 1 (maternal haplotype transmission was determined noninvasively in an autosomal dominant pattern), and 20 pregnancies with a male fetus with a 50% risk of Duchenne/Becker muscular dystrophy (maternal haplotype transmission was determined noninvasively in an X-linked recessive pattern). Gestational age at sampling ranged from 6 to 17 weeks of gestation (mean, 11 weeks).

Patient information is summarized in S3 Table.

### Optimal settings

To refine our pipeline, we tested the impact of several parameters on result quality using data from the training cohort.

#### Effect of the thresholds used to specify a heterozygous position in gDNA

We investigated two alternatives for defining heterozygous SNPs in gDNA. In the extensive definition, heterozygous SNPs in gDNA are defined by an allelic frequency of the alternative allele (AF) comprised between 0.15 and 0.85, whereas homozygous positions in gDNA are defined by an AF <0.15 or >0.85. In contrast, the restrictive definition corresponds to heterozygous SNPs in gDNA defined by an AF comprised between 0.35 and 0.65, while the AF of homozygous positions in gDNA remains unchanged at <0.15 and >0.85. An extensive definition of a heterozygous position could retain more informative positions than the restrictive definition but is likely to introduce SNP categorization errors due to misspecification of SNP positions associated with biased allele frequency. We investigated the effect of extensive versus restrictive definition of heterozygous SNPs in gDNA on the results by examining their impact on both scores (S1 Fig). As expected, the use of the restrictive definition leads to a reduction in the number of SNP4 that can be used for the eRHDO analysis of the training cohort, resulting in a lower $S_{b}$. On the other hand, this restrictive definition is associated with a significantly increased $S_{c}$, reflecting a marked improvement in the quality of the results (S4 Table). Thus, we decided to opt for the restrictive definition.

#### Effect of the minimum sequencing depth

Since a lower minimum sequencing depth threshold may retain information at more positions but is likely to be less accurate in determining allelic ratios, we explored different minimum thresholds for sequencing depth for our gDNA and cfDNA analyses, denoted by gDNA sequencing depth (NDP) and cfDNA minimum sequencing depth (PDP), tested at 8x, 15x and 30x, and 15x, 30x, 45x and 60x, respectively. We objectively examined their effect on $S_{c}$ and $S_{b}$. With PDP fixed, we found no difference in our scores with higher NDP (S2 Fig). Similarly, PDP variation did not affect the $S_{c}$ (S3 Fig), indicating no effect on the concordance of haplotype block classification between forward and reverse orientation. However, we found significant differences with respect to $S_{b}$ (*p*-value for Friedman test of 4.8 × *e*^−24^; S5 Table). The $S_{b}$ value increases at low PDP, reflecting a better quality of analysis: the smaller the minimum sequencing depth for calling a SNP4 in cfDNA, the larger the number of SNP4 considered in the eRHDO analysis, with potentially a larger the number of blocks. Therefore, we set the minimum sequencing depth for calling a SNP in cfDNA at 15x.

#### Effect of the minimum interSNP distance threshold

Previous reports have used a minimum interSNP distance threshold of 200 base pairs (bp) to minimize the risk of linkage between two SNPs and a resulting bias that would hinder allelic dosage analysis, but may also discard positions that would be informative [18, 19]. To assess this risk, we tested the effect of this threshold at 50 bp, 100 bp and 200 bp. The interSNP distance significantly affected $\;S_{b}$, with a *p*-value for the Friedman test of 3.6*e*^−46^, suggesting that an increased distance between two SNPs could result in a loss of information by reducing the number of events analyzed (S4 Fig and S6 Table). To maximize $S_{b}$, we chose a minimum distance of 50 bp between two SNPs to be considered.

#### Impact of statistical risks

When implementing the RHDO method, the statistical risks associated with the SPRT control the proportion of tolerated misclassification errors, and thus have a major impact on both $S_{c}$ and $S_{b}$. Permissive risks, i.e. risks closer to 1, facilitate the conclusion of the SPRT test, which obviously leads to smaller haplotype blocks and better associated $S_{b}$ (S5 Fig), but they have a variable impact on $S_{c}$ (S6 Fig). For low values of the parameters fetal fraction, number of SNPs and depth of sequencing, it is rather difficult to obtain conclusive results, and higher values of the statistical risks associated with smaller blocks help in this sense (S7 Fig). However, in reasonable situations, the misclassification errors (S8 Fig) induced by permissive statistical risks increase drastically. Therefore, we decided to set the two risks to 1/1000 in order to reduce the misclassification errors at most. Note that our pipeline allows to be adjusted to other values if deemed necessary.

In conclusion, the study of the training cohort allowed us to test different settings combinations, to objectively examine their impact on the quality scores, and to identify important quality control checkpoints. In fine, the optimal settings combination for test cohort exploration corresponded to the restrictive definition of heterozygous SNP, minimum NDP = 8x, minimum PDP = 15x, minimum interSNP distance = 50bp, and type I and type II error risks associated with SPRT = 1/1000.

### Quality assessment

Quality control points have been implemented at all stages of the workflow to minimize the risk of errors, but also to allow the users to assess the robustness and confidence they can have in their technical result.

#### Quality control tags associated with identity verification

After at-risk and non-at-risk haplotype determination, SNPs are classified into the previously described categories depending on the combination of parental genotypes’ [16]. We adapted the SNP categorization by considering the relationship of the proband to the couple and his status towards the familial SGD for subclassification, as well as his sex for X-linked transmission. Indeed, possible genotype combinations and decision thresholds may differ depending on whether the individual tested for parental haplotyping is a previous child or a relative of the couple. For quality purposes, we have also included two "WARNING” categories, as consanguinity, undetected miscarriage or undisclosed false paternity may confound interpretation. Previously named SNP5, where both parents are heterozygous and the at-risk haplotypes cannot be inferred, were named "WARNING_2” for "uninformative genotype combination”. Impossible genotype combinations were called "WARNING_1”, for example parents "AA” and "BB” with a first child as proband genotyped "AA”. This second category may be indicative of a sample swapping, incorrect assignment of proband relationship, or low-quality sequencing (S1 and S2 Tables).

#### Quality control tags associated with DNA capture and sequencing

Important Allele Dropout (ADO) can lead to erroneous determination of the fetal fraction, lower confidence in paternal inheritance, or hinder the determination of maternal haplotype ratios. Therefore, we return an evaluation of the proportion of ADO using SNP1 (fetal genotype AB and maternal genotype AA), as the proportion of fetal specific B allele not detected in maternal plasma (AF of B allele in maternal plasma = 0). Although this parameter does not block subsequent SGD-NIPD analyses, a significant ADO (>20% of SNP1 positions) should lead to a cautious interpretation of the result.

The sequencing error rate for each family was estimated using SNP2 density plots, corresponding to positions where both parents are homozygous for the same allele: the fetus is expected to be homozygous as well and any other allele found at the locus can be considered a sequencing error. Again, a significant sequencing error rate associated with one or more samples (>2%) should result in a cautious interpretation of the SGD-NIPD result.

#### Quality control tags associated with fetal fraction evaluation

The fetal fraction was estimated without prior knowledge of the SNP category, using the minor allele frequency distribution (MAF). In its density plot, the first peak is expected to correspond to the frequency of the fetal-specific allele at positions where the fetus is heterozygous in a homozygous maternal background, allowing the fetal fraction to be inferred from twice this haploid frequency. Estimation using the SNP1 distribution, where both parents are homozygous for different alleles, was retained for comparison purposes. The fractional fetal DNA concentration is evaluated using the formula *f* = 2*p*/(*p* + *q*) where p is the number of sequenced reads from the fetal-specific allele and q is the number of sequenced reads from the maternal and fetal common allele.

### Test cohort results

Patient informations and eRHDO-based SGD-NIPD results are shown in S3 Table.

The sequencing error rate was consistently low across our families, ranging from 0.3% to 1.4% (mean, 0.68%), consistent with the sequencing error profile of the platform [25]. Although we chose not to set a fixed threshold for interpretation, an elevated sequencing error rate should alert to the risk of a low-quality result secondary to degraded DNA or a technical issue during DNA extraction or sample processing and sequencing.

The fetal fraction estimated from the distribution of all sequenced positions (minor allele frequency, MAF), as described in supplemental data, ranged from 2.9 to 19.3% (mean 9.3%). Three major discrepancies were found between MAF-based determination and SNP1-based determination, related to an abnormal distribution of the fetal-specific SNP1 allele frequency in maternal plasma caused by major ADO in these three samples (families 2_29, 2_33, 2_40).

**Fig 3:**
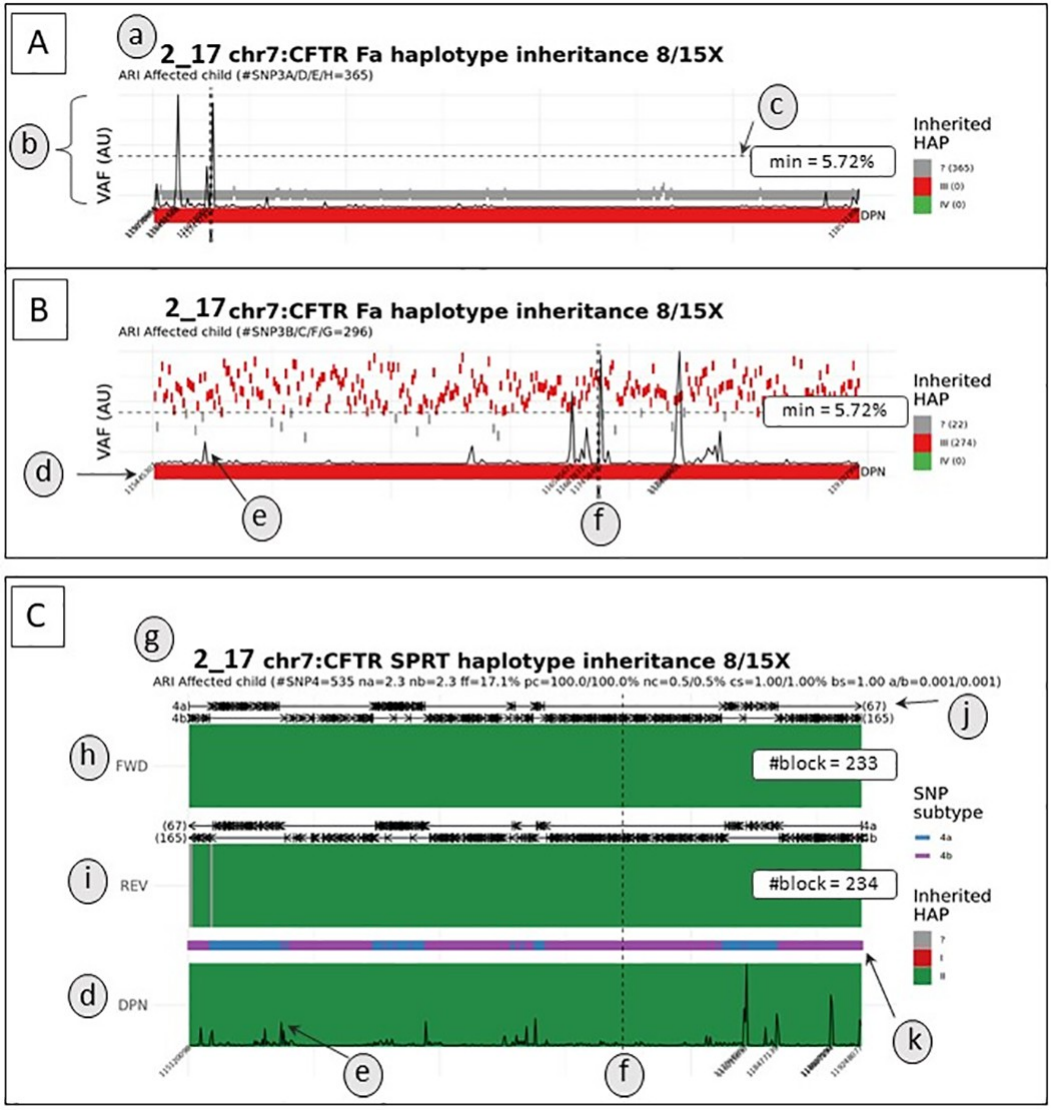
Example of a graphical result for autosomal inheritance in a family at risk of transmitting cystic fibrosis (family 2), subdivided into paternal (A, B) and maternal (C) inheritance. In this example, SNP3A/D/E/H correspond to SNP3 located on the “non-at-risk” paternal haplotype (A), while SNP3B/C/F/G correspond to SNP3 located on the “at-risk” paternal haplotype (B). “Non-at-risk” and “at-risk” haplotypes are shown in green and red, respectively. (a) For each result, the input data are summarized as follows: family ID, tested locus and parental inheritance, gDNA/cfDNA minimum sequencing depth, transmission mode (ARI = autosomal recessive inheritance, ADI = autosomal dominant inheritance, RXI = recessive X-linked inheritance) and number of SNP3. (b) Qualitative detection of fetal-specific SNPs with an allelic frequency (VAF) higher than our threshold visualized in (c) (AU = arbitrary units). SNP3A/D/E/H from the “non-at-risk haplotype” were not detected in cfDNA (top), whereas the majority of SNP3B/C/F/G from the “at risk” haplotype were qualitatively detected above the background threshold (bottom). (d) Fetal genotype from sequencing of fetal gDNA obtained by invasive sampling (e) Plots of inter-SNP distance. Each peak indicates a longer genomic distance between two consecutive tested positions. (f) Location of the parental mutation, symbolized by a vertical dashed line. (g) Input data and quality parameters of the SPRT analysis, namely number of SNP4, mean number of SNPs 4α or 4β per block (na and nb), concordance between conclusive haplotype blocks in forward and reverse orientation (pc) and proportion of nonconclusive haplotype blocks (in gray) among all blocks (nc), and type I and type II statistical errors used in the SPRT test (a/b). (h) and (i) SPRT analysis in forward and reverse orientation, respectively. (j) Visualization of haplotype blocks, divided into 4α (top) and 4β (bottom) analyses, with number of conclusive blocks. (k) Distribution of SNPs 4α (blue) and 4β (purple) along the genomic region.

An example of our result visualization is shown in Fig 3.

Paternal inheritance was determined only in families at risk for recessive cystic fibrosis (CF). All results were conclusive and concordant with the fetal genotype obtained by invasive sampling except one. In family 2_12, a recombination occurred close to the paternal variant location and the transmitted paternal haplotype at this position could not be inferred. Although the mother was a carrier of a different pathogenic variant, because this position was not targeted by our capture panel, the paternal variant could not be qualitatively detected by direct visualization of the paternal-specific allele on the bam sequence files.

**Fig 4:**
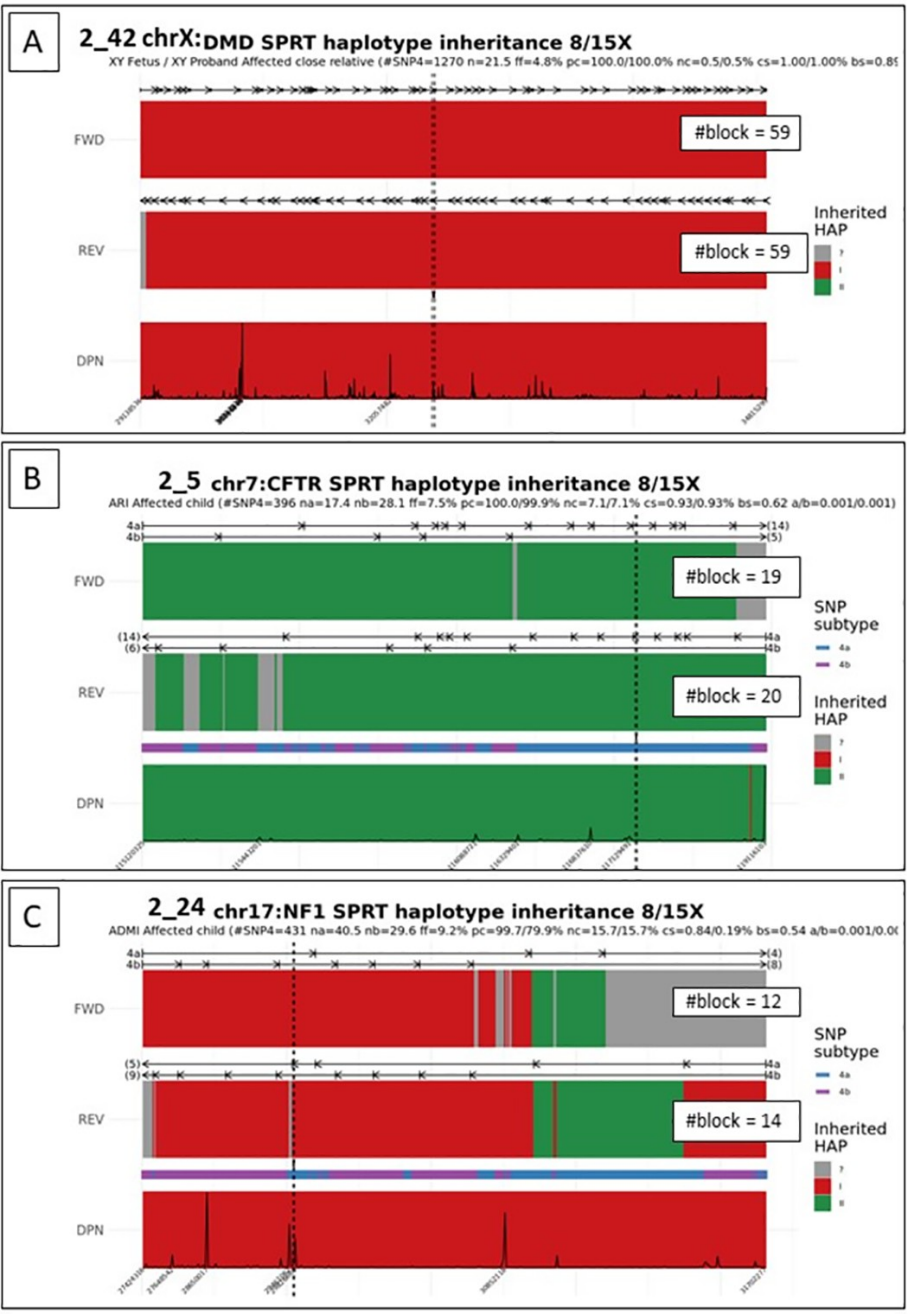
Examples of results with different quality scores. (A) Family 2_42 at risk of transmitting Duchenne/Becker muscular dystrophy. A high number of tested SNP4 (n = 1270) combined with a deep sequencing depth (mean = 211X) allowed the detection of a large number of blocks (59 haplotype blocks in forward direction and 59 haplotype blocks in reverse direction) with a high concordance in favor of the transmission of HapI (Sc = 1.00), despite a relatively low fetal fraction (f = 4.8%). Sb was estimated to be 0.89. (B) Family 2_5 at risk of transmitting cystic fibrosis. This family presented with a reasonable number of SNP4 (n = 396) and fetal fraction (f = 7.5%), but only a few haplotype blocks could be reconstructed (n = 39 blocks in forward + reverse directions) with a relatively high number of SNP4 per block (na = 17, nb = 28.1), resulting in a lower block score (Sb = 0.62). Note that the high concordance between forward and reverse analysis (Sc = 0.93), as well as the position of the maternal variant along the CFTR locus (black dashed line), still allow to conclude in favor or the transmission of HapII with high confidence. (C) Family 2_24 at risk for maternal transmission of neurofibromatosis type I. Only 26 haplotype blocks could be defined with a high mean number of SNPs per block (na = 40.5, nb = 29.6), resulting in longer blocks and therefore a lower block score (Sb = 0.54). Since 2/26 blocks were incorrectly classified as HapII instead of HapI, suggesting a SPRT error, the concordance score was estimated to be 0.84. Although the maternal variant is located in a concordant, HapI region, the small number of haplotype blocks, the haplotype change at the 3’ end of the locus, and the unclassified blocks at both ends prevent us from concluding on the fetal status for NF1. In a diagnostic setting, the analysis could be repeated on a subsequent sample. Sb: Bloc Score; Sc: Concordance score; na: mean number of SNP4a per haplotype bloc; nb: mean number of SNP4b per haplotype bloc; f: fetal fraction.

Considering maternal inheritance, 59 analyses were performed on 56 samples, as one family (family 2_26) was included in our study three times during three consecutive pregnancies and the cfDNA of one given pregnancy was analyzed twice using either PND’s gDNA for parental haplotype inference. 56/59 samples were conclusive (94.9%) and concordant with the fetal genotype obtained by invasive sampling. Of these, 43/59 results (72.9%) were conclusive with high quality results. All of these high quality results had a $S_{c}$ and a $S_{b}$ greater than 0.7 (Fig 4A). 13/59 (22%) were conclusive and concordant with the fetal genotype, but with slightly lower quality results. As suggested by our computer simulations, these low-quality results were probably due to a small number of events tested, either because of low cfDNA sequencing depth, consistently less than 100x (meaning a small number of events counted at each locus), or low fetal fraction (families 2_33 and 2_40) (meaning a small allelic imbalance to be detected). For these families, the block score $S_{b}$ was between 0.55 and 0.7 (Fig 4B). Finally, 3 samples were inconclusive (5.1%). In family 2_39, a recombination event occurred in the block adjacent to the position of the maternal variant, which did not allow confident haplotype inference at this position (Fig 4C). Family 2_12 had a low fetal fraction and a low total cfDNA sequencing depth, so that not enough positions could be examined. Similarly, not enough SNP4 were included in the RHDO analysis of family 2_14 beacause few SNP4 were identified in the *CFTR* locus and the sample returned with a relatively low fetal fraction (5.9%).

We suggest that the use of user-defined thresholds could help users when interpret their results. In our hands, we observed that scores >0.7 were associated with high quality results, while scores <0.55 were associated with poor quality results.

### Computer simulation for quantification of the respective effect of quality-influencing factors

Based on the study of training and test cohorts, we selected biological and analytical critical factors that appear to be crucial for the quality of the analysis, namely fetal fraction, number of SNP4 available for eRHDO analysis, and depth of cfDNA sequencing. To evaluate their respective relevance, we simulated sequencing data on which we tested the quality scores. In each simulation, one of the parameters was changed with the statistical risks fixed at 1/1000.

As shown in S9 Fig, these three parameters were confirmed to have a strong influence on the quality of the results: the higher, the easier the conclusions of the SPRT tests, and the smaller and the more conclusive the blocks, the higher the $S_{c}$ and $S_{b}$ values.

To quantify the effect of all these parameters on the quality of the results, we also performed a sensitivity analysis by calculating of Sobol sensitivity indices. In short, these indices measure how much of the variance in the model output is due to each parameter to be fixed. A low sensitivity index (close to 0) reflects that variations in the associated parameter will lead to small variations in the results, while a high sensitivity index (close to 100) implies large changes. Sensitivity analysis also provides an opportunity to explore the interaction effects of parameters on the model. Finally, in addition to the calculation of Sobol indices, we finally performed ANalysis Of VAriance (ANOVA) tests to identify the statistically significant effects of the parameters on both outcomes. The Sobol indices of fetal fraction, number of SNPs, sequencing depth, their combinations of them and the corresponding ANOVA *p*-values are shown in S7 Table.

These further analyses confirm the strong effect of the fetal fraction on both $S_{c}$ and $S_{b}$. The effect of the number of SNPs and the sequencing depth, although still highly significant, is less strong. Only the interactions between the parameters, including the fetal fraction, lead to significant variations in $S_{c}$, again emphasizing its importance. On the contrary, $S_{b}$ is sensitive to simultaneous changes of all parameters.

## Discussion

Since the initial description of RHDO, no practical recommendations or guidelines have been proposed for data generation and analysis, fetal fraction assessment, quality thresholds, and interpretation [26]. In this report, we attempted to develop a standardized workflow for clinical implementation of a reliable SGD-NIPD. To this end, we worked on each step of the previously described RHDO and optimized this workflow on a training cohort, composed of families in which fetal gDNA was used to infer parental haplotypes. We then tested it on a test cohort, as close to a clinical setting as possible, with different modes of transmission and different types of molecular abnormalities, as well as multiple proband and couple familiy schemes.

Since our goal is to provide the most direct computational analysis, we tried to consider every situation that might arise in genetic counseling in our workflow construction. We detailed each SNP category, taking into account the relationship of the proband to the couple (previous child or close relative) as well as his status with respect to the parental variants (affected/carrier or unaffected). This allows haplotype phasing and decision threshold calculations to be performed automatically, without the need to customize the informatics analysis pipeline for specific situations.

A key point of our approach is that our workflow is designed to allow users to adjust several settings at different steps of the process, according to their needs. The study of the training cohort, as well as simulated data analysis, allowed different values for these variables to be tested to objectively examine their impact on the quality scores. The extensive or restrictive thresholds used to specify a heterozygous position in gDNA, the minimum sequencing depth of cfDNA, the minimum interSNP distance, and the statistical risks associated with SPRT were investigated and adjusted.

Quality control points have been implemented at all stages of the workflow, to minimize the risk of error, but also to allow users to assess the robustness and confidence they can have in their results. During SNPs categorization, we added two "WARNING” categories for uninformative and impossible genotype combinations. The former can help expand the range of family members that can be tested, while the latter will be useful in identifying the potential causes of an unusual result. For example, too many SNPs excluded from the analysis because of uninformative combinations may explain an inconclusive result on qualitatively correct experimental data that might reflect the analysis of a consanguineous parental couple. An overrepresentation of impossible combinations may indicate an error in the input data for the sequencing analysis, a sample swap, or contamination during the technical steps.

Because ADO can interfere with the analysis or even cause a false result, we added an assessment of the mean ADO rate based on the SNP1 analysis to the output data. Finally, the sequencing error rate density was estimated for each family using SNP2, corresponding to positions where both parents are homozygous for the same allele. A significant ADO or sequencing error rate should lead to a cautious interpretation of the result.

The main quality-influencing factors are the fetal fraction, the number of SNP4 tested and the depth of cfDNA sequencing, which affects the number of events tested. Computer simulations allowed the identification of the fetal fraction as the most dominant of these factors. Since the number of SNP4 depends on the combination of parental genotypes and the fetal fraction is biologically determined in a given cfDNA sample, we recommend that sequencing conditions should be optimized in order to obtain sufficient cfDNA sequencing depth, higher than 100x in this study.

Because the fetal fraction must be accurately assessed, we chose to use the minor allele frequency distribution of all the SNPs targeted in the capture panel, rather than relying on prior knowledge of their category, as is done for SNP1-based assessment. By increasing the number of events, we hope to obtain a more accurate estimate of the fetal fraction than the SNP1-based evaluation.

Paternal inheritance determination relies on the qualitative detection of fetal-specific SNPs that are present at a very low allelic fractions in maternal plasma. We introduced a minimum allelic fraction threshold for considering a SNP3 as significantly detected, depending on the sequencing error rate and the fetal fraction of the sample, to discriminate between background noise and SNPs that were actually transmitted to the fetus. We also divided the analysis into "non-at-risk haplotype detected" and "at-risk haplotype detected" and required concordance between these two analyses.

Finally, we designed our output to be as simple and unambiguous as possible, while retaining as much information as possible. To this end, we included a graphical visualization that summarizes the input data, reports the result with a two-color code, and includes several quality parameters to facilitate biological interpretation. To account for the risk of misclassification due to an undetected recombination event, we plotted the distance between two neighboring SNPs included in the analysis as a black curve in this final visualization, as the risk of recombination between two loci increases with genomic distance [27, 28].

Our workflow proved to be very robust, as we obtained 94.9% conclusive and correctly inferred fetal genotypes in our real-life-like cohort, with no false negatives or false positives (56/59 conclusive and correctly inferred cases + 3/59 inconclusive cases). Rather, our quality controls proved to be stringent enough that low quality analyses returned inconclusive results, mostly in the situation of an insufficient number of tested events, either because of a low fetal fraction, low sequencing depth, or because of a small number of informative SNPs in the families. Our objective quality scores consistently supported our biological interpretation, being higher than 0.7 for high-quality analyses and lower than 0.55 for low-quality analyses. However, we chose not to set a threshold for analysis, as we developed these quality scores to support interpretation rather than as strict quality criteria. In fact, they refer only to the quality of the haplotype identification in maternal plasma and do not take into account the position of the variant along the target locus. They could therefore be close to 1 in a family where no SNPs 5’ or 3’ of the variant can be identified, a situation in which the maternal haplotype at the variant’s locus cannot be determined with certainty because a recombination event cannot be excluded. Similarly, if a recombination event is identified near the parental variant position, the analysis may be of overall high overall quality, but the interpretation of the SGD-NIPD should be inconclusive, regardless of the quality scores.

This recombination risk is a well-described limitation of the MPS-based NIPD. Because it relies on an indirect approach, the occurrence of a recombination event close to the parental variant position may not allow to conclude [23]. While this limitation cannot be fully addressed, we hoped to aid interpretation by adding information on the parental variant position along the haplotype. In our test cohort, we observed a total of 7 recombination events, as expected from the known recombination frequencies in the genomic regions studied. Five of them occurred at sufficient genomic distance from the parental variant and did not affect the SGD-NIPD result, but the other two confounded the SGD-NIPD interpretation. Note that while a recombination event on the maternal allele close to the familial variant in the context of maternal autosomal dominant or X-linked transmission would not allow NIPD, if both parents carry different pathogenic variants in an autosomal recessive setting, the paternal allele can be searched directly in maternal cfDNA sequences in a qualitative manner.

Another major limitation of haplotype-based SGD-NIPD is the need for genotype information from the proband for haplotype phasing. Care must be taken in the selection of this proband. In some cases, one of the parents and their unaffected sibling may not share a haplotype. Similarly, when parents carry the same variant in an autosomal recessive disorder, the parental origin of the variant in a heterozygous first child will most likely be unknown, unless an indirect technique has been previously performed in these families. The parental haplotypes will be correctly phased but identification of the at-risk and non-at-risk haplotypes will not be possible. Although we have anticipated most situations, this workflow cannot be offered to a family in which a variant has arisen *de novo*, or if no other family member is available for testing. While *de novo* haplotyping methods have been reported in the context of NIPD [29–34], experimental approaches often require specialized equipment and reagents, making them costly and inappropriate for implementation in a clinical setting. Computational approaches, while accurate and less expensive, may require specific bioinformatics skills and rely on a population database, making them unreliable for rare or private variant phasing [35, 36]. An exception would be the Targeted Locus Amplification approach, described by Vermeulen et al., which does not depend on any specific equipment but may still require adaptations for implementation in laboratories unfamiliar with chromosome conformation capture techniques [37]. However, experience in preimplantation genetic testing has shown that refusal of a referral due to inability to analyze a proband rarely occurs [38].

MPS-based techniques also require a careful study of the genomic environment of the tested gene before designing the enrichment panel. In our study, we tested hemophilia A carriers in our training cohort, but had to exclude them from our test cohort, because our capture probes did not target enough SNPs at the 3’ end of the *F8* gene, which is located at the telomeric end of the X chromosome. Specific adaptations may be required for certain disorders, such as increasing the number of targeted SNPs at the locus of interest by lowering the minor allele frequency threshold used in the capture probe design, or increasing the size of the target region around the gene.

With the widespread implementation of non-invasive prenatal testing for fetal aneuploidy, incidental maternal findings have been reported, particularly maternal malignancy [39, 40]. To our knowledge, this situation has not been reported in the context of NIPD for SGD. The occurrence of an abnormal profile of our density plot of MAF for fetal fraction estimation may suggest an underlying maternal disorder. To date, disclosure of results suggesting maternal malignancy remains controversial [40, 41], but the possibility of maternal incidental findings during cfDNA analysis should be mentioned during pretest counseling.

In conclusion, we report an easy-to-use workflow for SGD-NIPD. We have adapted the previously published relative haplotype dosage analysis approach into a straightforward, easy-to-perform workflow. Although thresholds for statistical risks and quality parameters can be easily adjusted depending on biological expertise, we propose an optimized value for each parameter. The accuracy and reliability of the entire process was then validated on a large real-life-like cohort. By testing multiple transmission modes, variant types, and proband relationships, we hope to review a wide range of situations commonly encountered in the context of PND. Future work will explore the cost of our approach compared to current clinical practice before this safe, non-invasive approach can be readily implemented as an accredited diagnostic service in a public health laboratory.

## Supporting information

S1 Fig Effect of the thresholds used to specify a heterozygous position in gDNA (extensive versus restrictive) on the block and concordance scores. In the extensive definition, heterozygous SNPs in gDNA are defined by an allelic frequency of the alternative allele (AF) comprised between 0.15 and 0.85, while homozygous positions in gDNA are defined by an AF <0.15 or >0.85. By contrast, the restrictive definition corresponds to heterozygous SNPs in gDNA defined by an AF comprised between 0.35 and 0.65 while homozygous positions’ AF in gDNA remain unchanged. An extensive definition of a heterozygous position could retain more informative positions than the restrictive definition but is likely to introduce SNP categorization errors due to misspecification of SNP positions associated with biased allele frequency.
(TIF)

S2 Fig Effect of NDP on the block and concordance scores (on rows) for different values of PDP (on columns). At PDP fixed, there is no difference in scores with higher NDP. Therefore, the minimal sequencing depth for calling SNP in genomic DNA was fixed at 8x.
(TIF)

S3 Fig Effect of PDP on the block and concordance scores (on rows) for different values of NDP (on columns). At NDP fixed, PDP variation did not impact the Sc, indicating no impact on the concordance of haplotype blocks classification between forward and reverse orientations. However, Sb value rises at low PDP, which reflects a better quality of analysis. Therefore, the minimal sequencing depth for calling SNP in cfDNA was fixed at 15x.
(TIF)

S4 Fig Effect of the interSNP distance on the block and concordance scores. (TIF)

S5 Fig Effect of the statistical risks associated with the SPRT on the block score for different values of the parameters (fetal fraction, sequencing depth and number of SNPs). (TIF)

S6 Fig Effect of the statistical risks associated with the SPRT on the concordance score for different values of the parameters (fetal fraction, sequencing depth and number of SNPs). (TIF)

S7 Fig Effect of the statistical risks associated with the SPRT on the percentage of non-concordance for different values of the parameters (fetal fraction, sequencing depth and number of SNPs). (TIF)

S8 Fig Effect of the statistical risks associated with the SPRT on the percentage of concordance score for different values of the parameters (fetal fraction, sequencing depth and number of SNPs). (TIF)

S9 Fig Effect of the parameters (fetal fraction, sequencing depth and number of SNPs) on the block and concordance scores. (TIF)

S1 Table SNP categorization for autosomal inheritance, taking into account the proband’s relationship to the pregnant couple and his status towards the familial pathogenic variants. (PDF)

S2 Table SNP categorization for X-linked inheritance, taking into account the fetal gender, the proband’s gender, his relationship to the pregnant couple and his status towards the familial pathogenic variants. (PDF)

S3 Table Patients’ information and NIPD results. ADMI = autosomal dominant maternal inheritance; ADPI = autosomal dominant paternal inheritance; ARI = autosomal recessive inheritance; RXLI = X-linked inheritance; AC = affected child; UC = unaffected child; AR = affected close relative; UR = unaffected close relative; DP = depth of sequencing; #SNP4 = SNP4 count; #FWD and #REV = halotype block count in either orientation; #MUA, #MUB, #MUX: mean count of SNP4α, SNP4β or SNP4 (X-linked inheritance); CS: concordance score; BS: block score; Preg = pregnancy number; Inh = genetic inheritance mode; Prob = proband.
(PDF)

S4 Table p-values obtained after testing differences between extensive/restrictive discrimination of heterozygous position in gDNA for both scores using Wilcoxon tests. The p-values are adjusted for multiple comparisons.
(PDF)

S5 Table p-values obtained after testing differences between each pair of variables PDP for each value of NDP using Wilcoxon tests. The global p-value (Friedman test) is indicated in brackets on the top of the table. All p-values are adjusted for multiple comparisons.
(PDF)

S6 Table p-values obtained after testing differences between each pair of variables (interSNP distance) for each value of the number of SNPs using Wilcoxon tests. The global p-value (Friedman test) is indicated in brackets on the top of the table. All p-values are adjusted for multiple comparisons.
(PDF)

S7 Table Sobol indices indicating the effect of all parameters (fetal fraction, number of SNPs and sequencing depth) and their combinations on the results quality (ISb and ISc for block and concordance scores respectively). p-values for ANOVA tests are indicated in brackets.
(PDF)

S1 File (PDF)
