# Supplementary figures and images for: Non-invasive prenatal diagnosis of single gene disorders with enhanced relative haplotype dosage analysis for diagnostic implementation
Source: PLoS One. 2023 Apr 24;18(4):e0280976. doi: 10.1371/journal.pone.0280976 (PMC10124834; doi:10.1371/journal.pone.0280976)

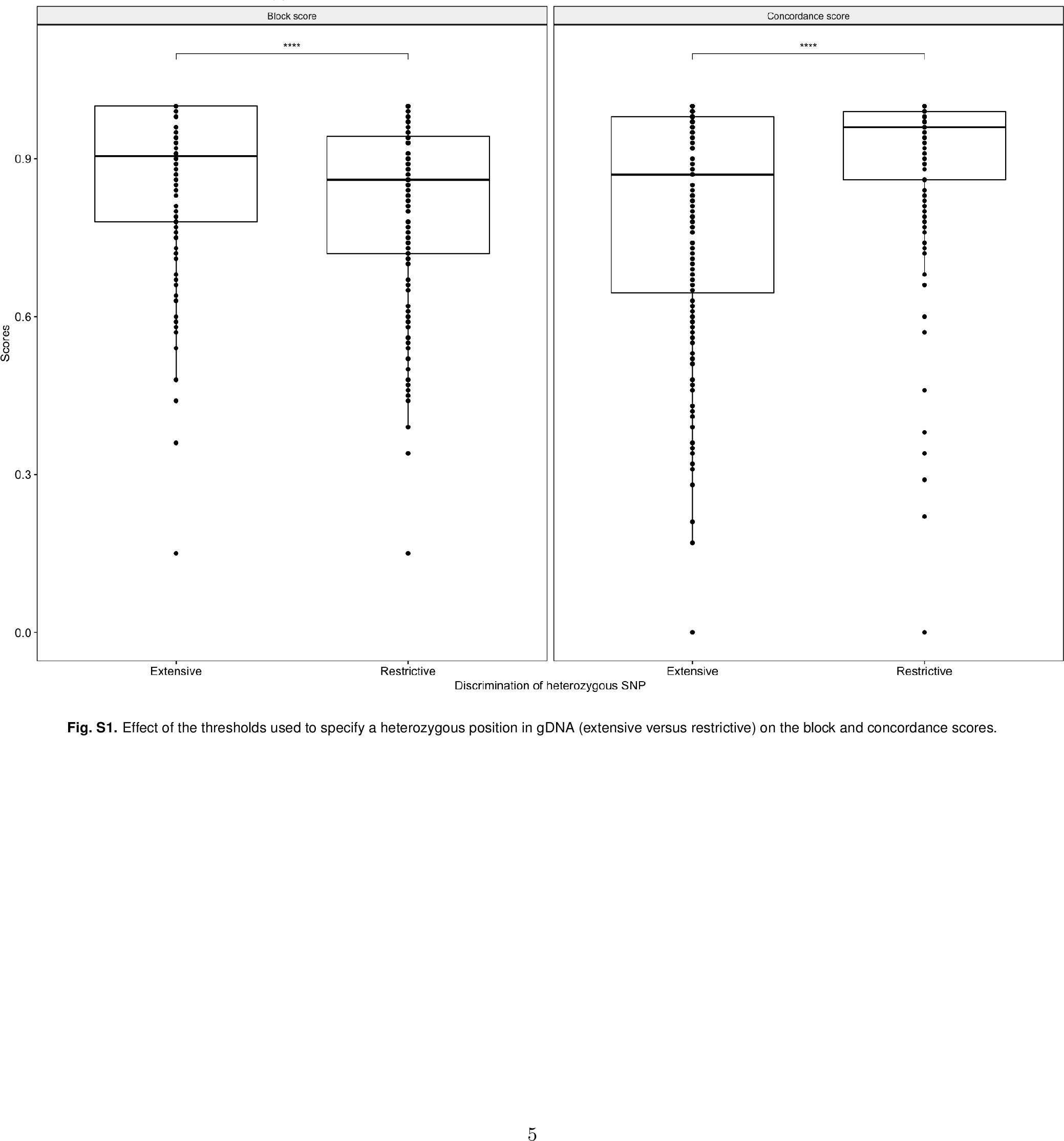

Supplement: S1 Fig — In the extensive definition, heterozygous SNPs in gDNA are defined by an allelic frequency of the alternative allele (AF) comprised between 0.15 and 0.85, while homozygous positions in gDNA are defined by an AF <0.15 or >0.85. By contrast, the restrictive definition corresponds to heterozygous SNPs in gDNA defined by an AF comprised between 0.35 and 0.65 while homozygous positions’ AF in gDNA remain unchanged. An extensive definition of a heterozygous position could retain more informative positions than the restrictive definition but is likely to introduce SNP categorization errors due to misspecification of SNP positions associated with biased allele frequency. (TIF) [file pone.0280976.s001.tif]

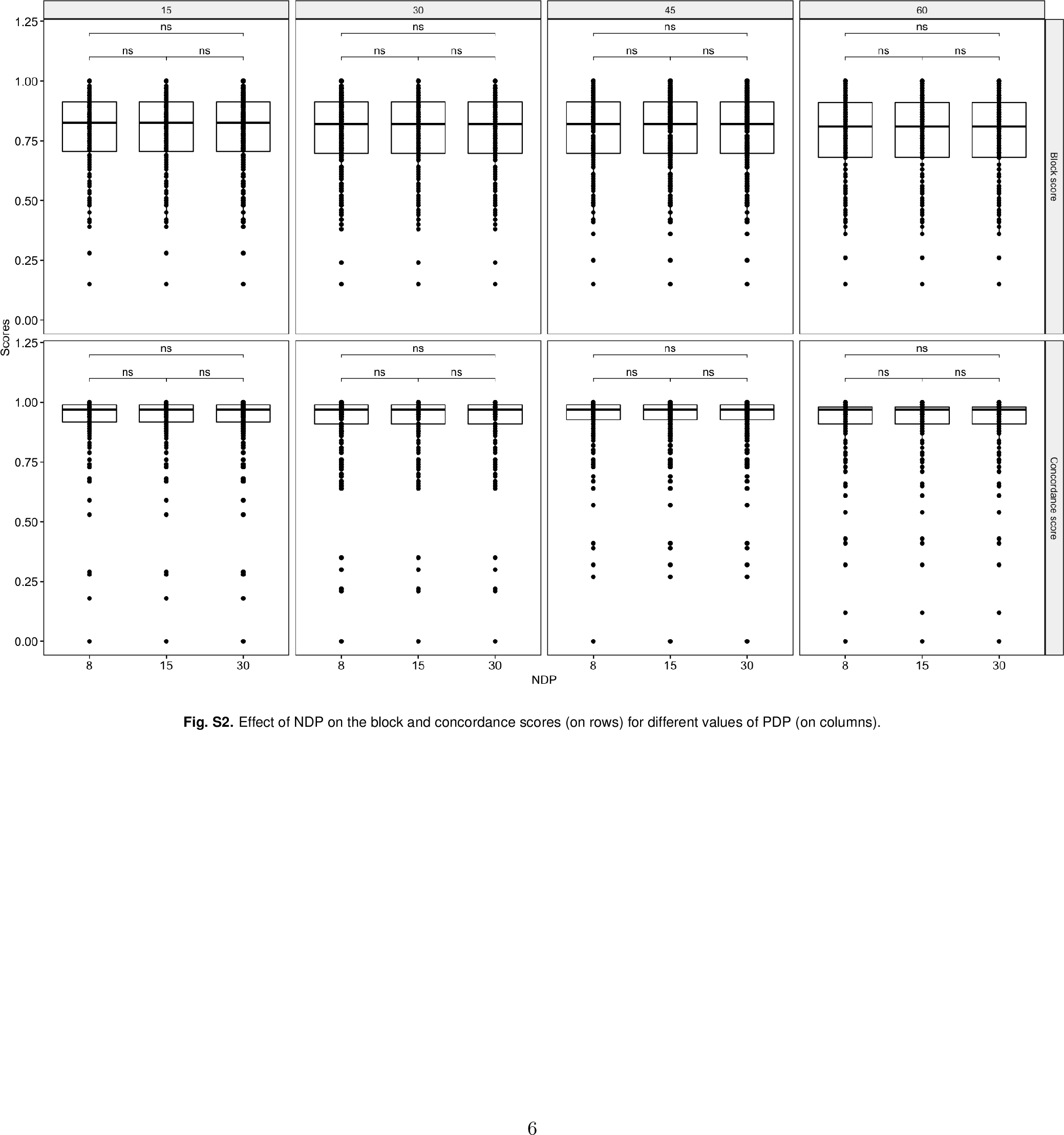

Supplement: S2 Fig — At PDP fixed, there is no difference in scores with higher NDP. Therefore, the minimal sequencing depth for calling SNP in genomic DNA was fixed at 8x. (TIF) [file pone.0280976.s002.tif]

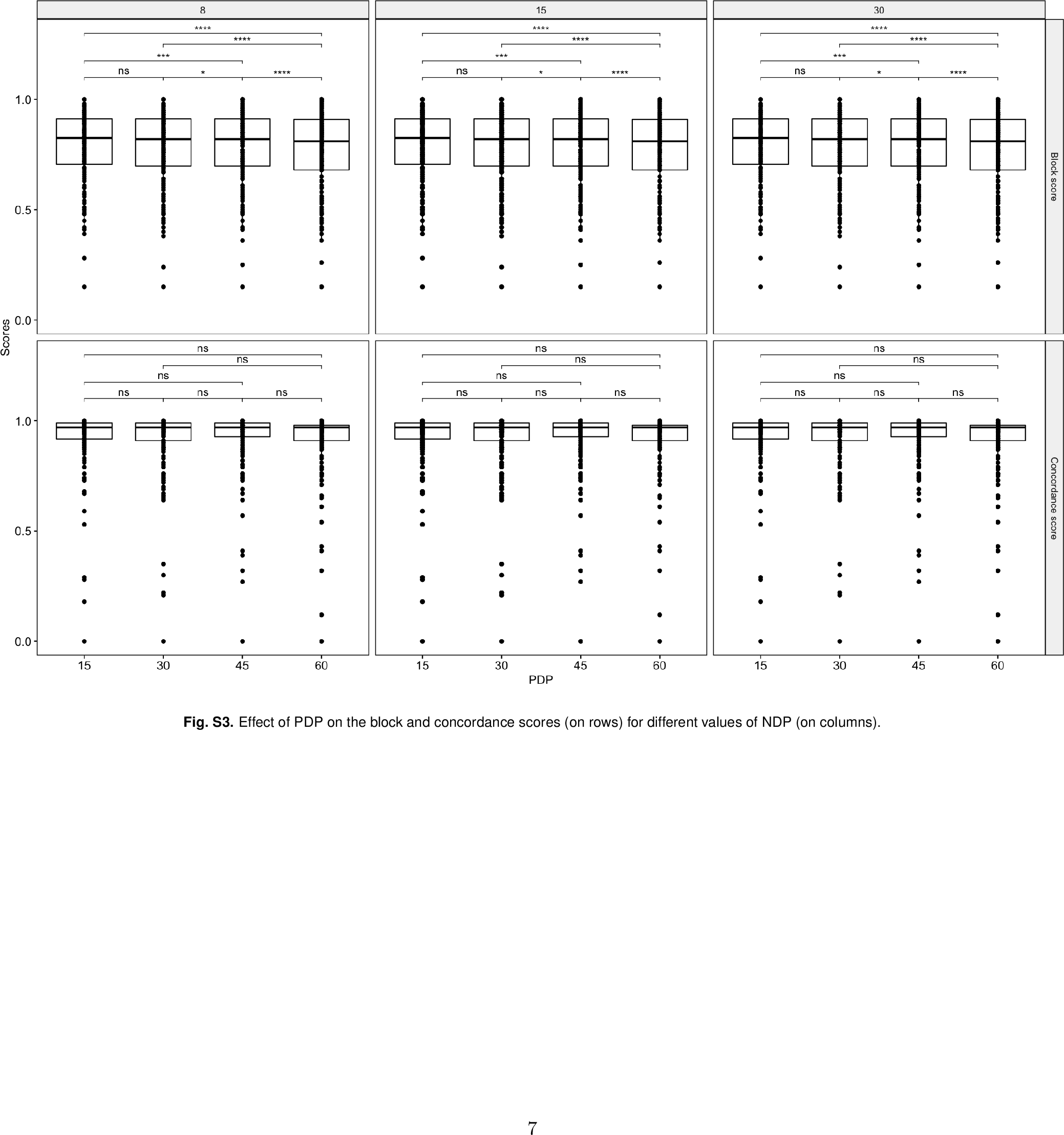

Supplement: S3 Fig — At NDP fixed, PDP variation did not impact the Sc, indicating no impact on the concordance of haplotype blocks classification between forward and reverse orientations. However, Sb value rises at low PDP, which reflects a better quality of analysis. Therefore, the minimal sequencing depth for calling SNP in cfDNA was fixed at 15x. (TIF) [file pone.0280976.s003.tif]

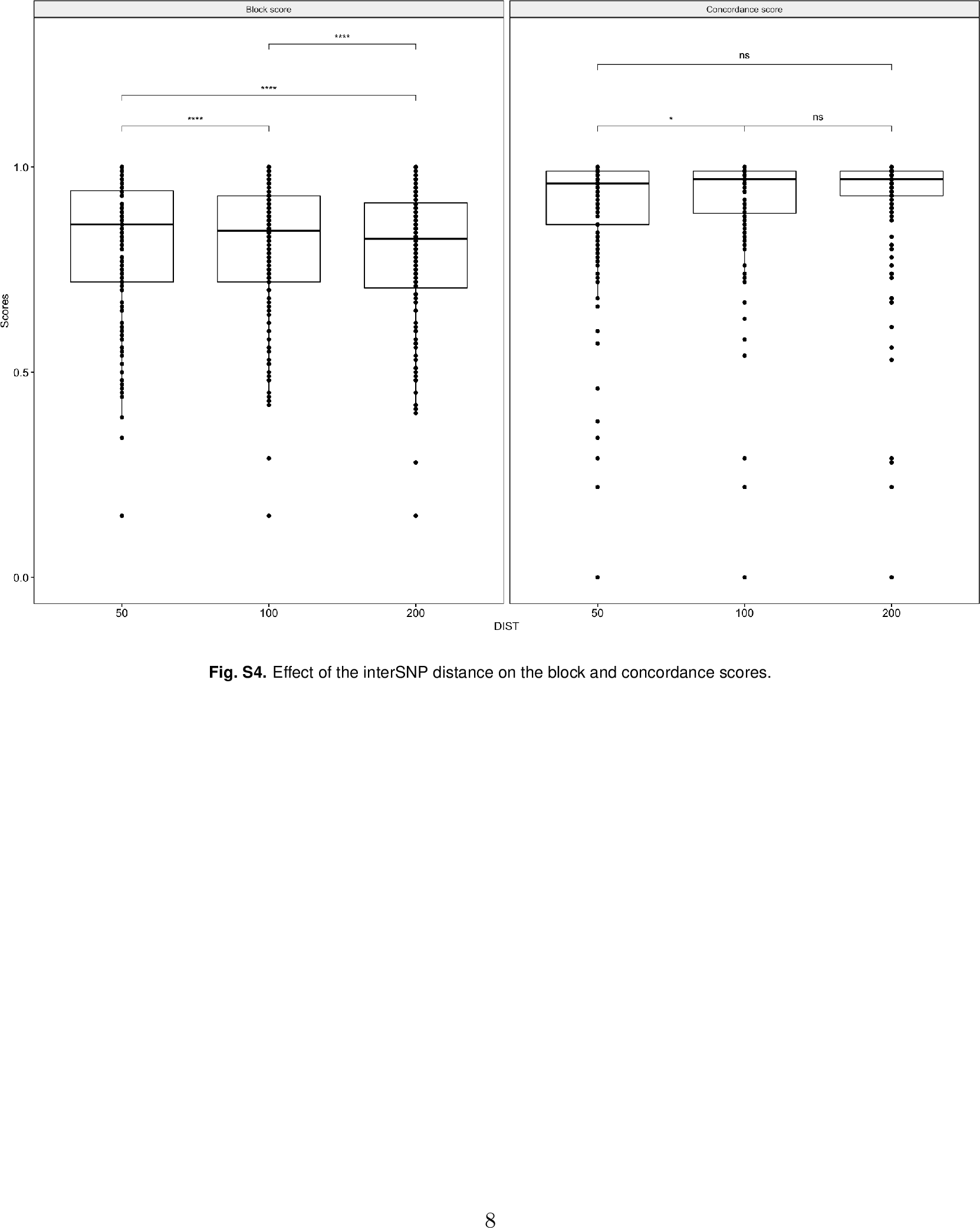

Supplement: S4 Fig — (TIF) [file pone.0280976.s004.tif]

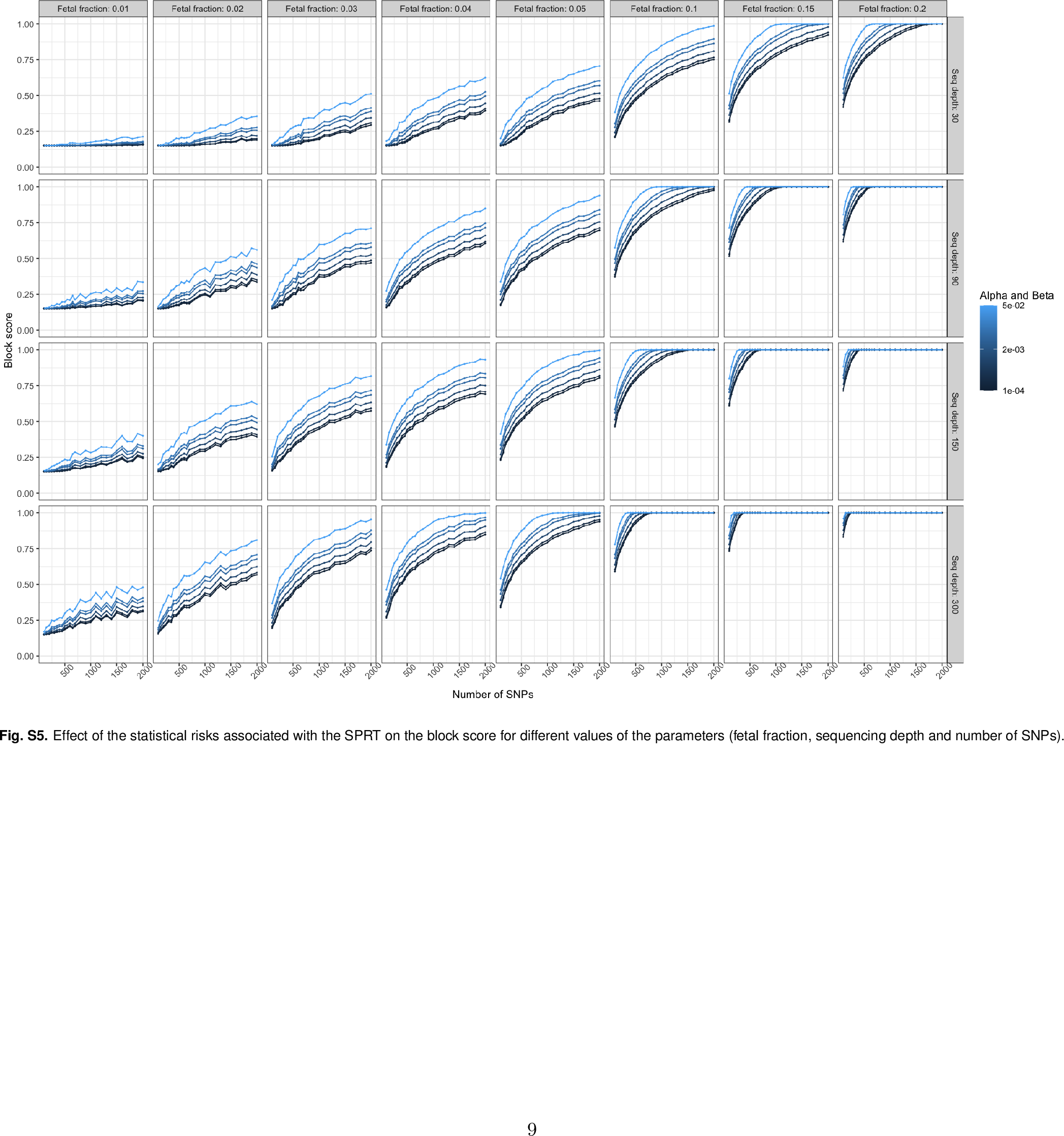

Supplement: S5 Fig — (TIF) [file pone.0280976.s005.tif]

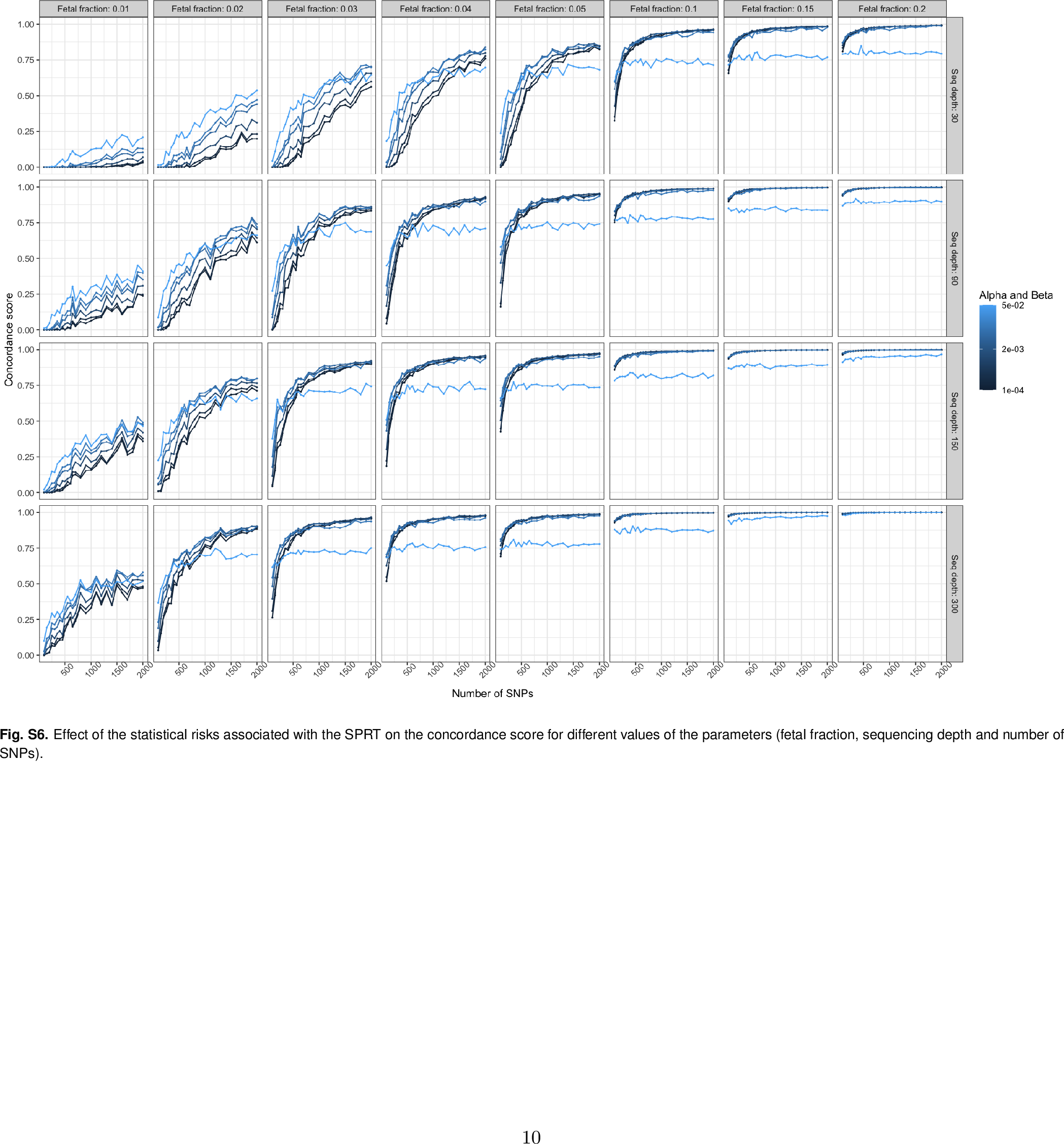

Supplement: S6 Fig — (TIF) [file pone.0280976.s006.tif]

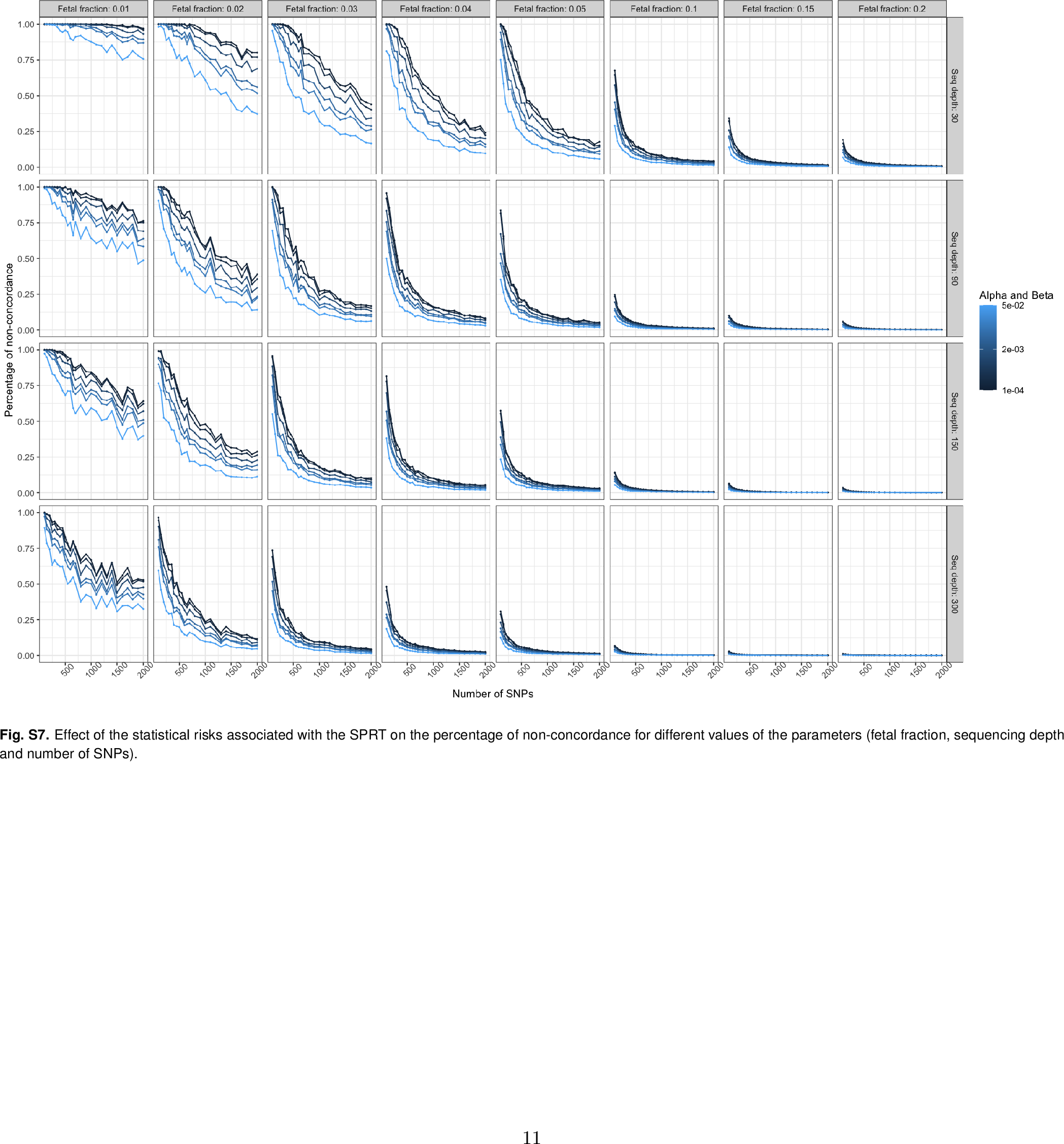

Supplement: S7 Fig — (TIF) [file pone.0280976.s007.tif]

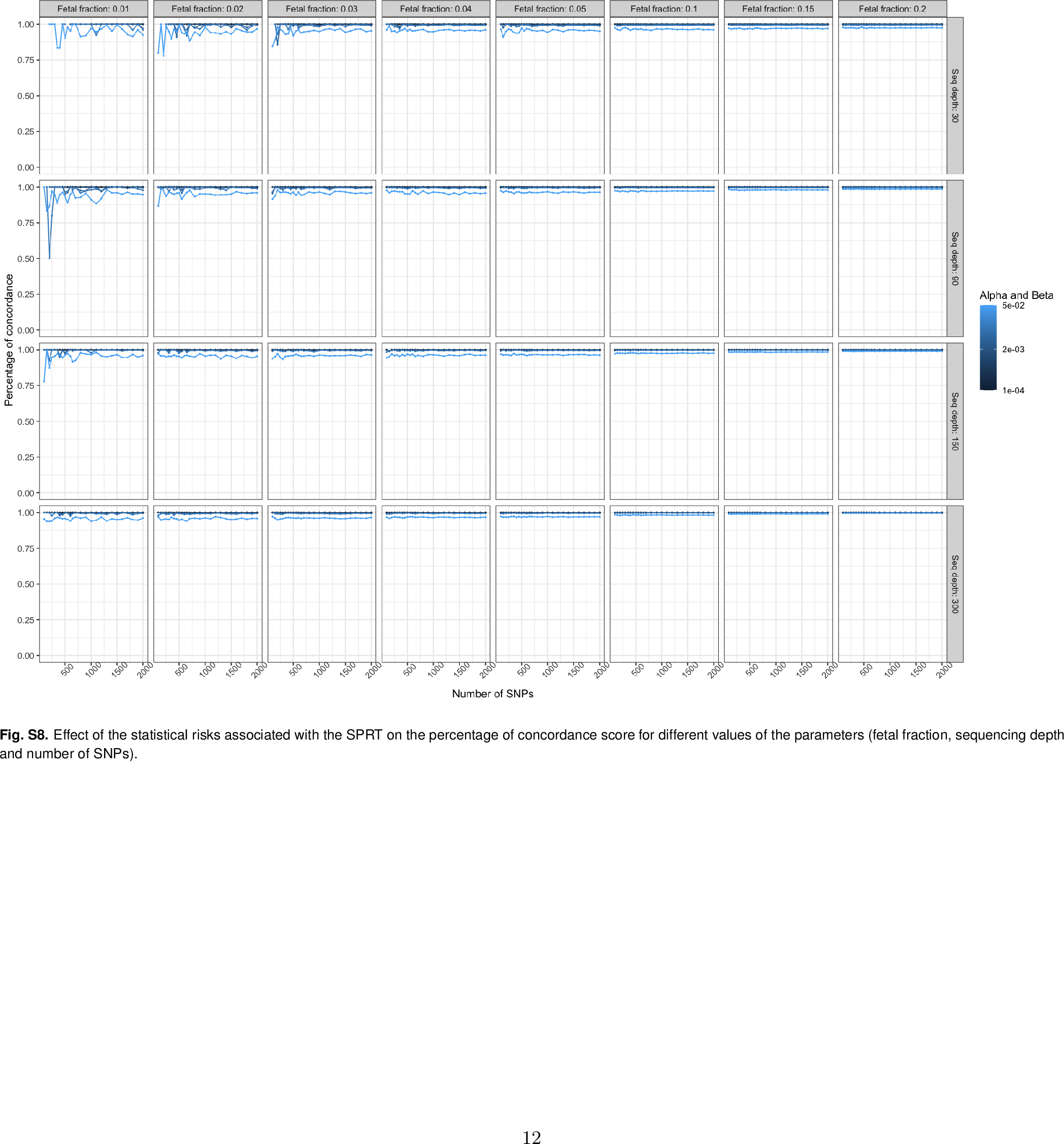

Supplement: S8 Fig — (TIF) [file pone.0280976.s008.tif]

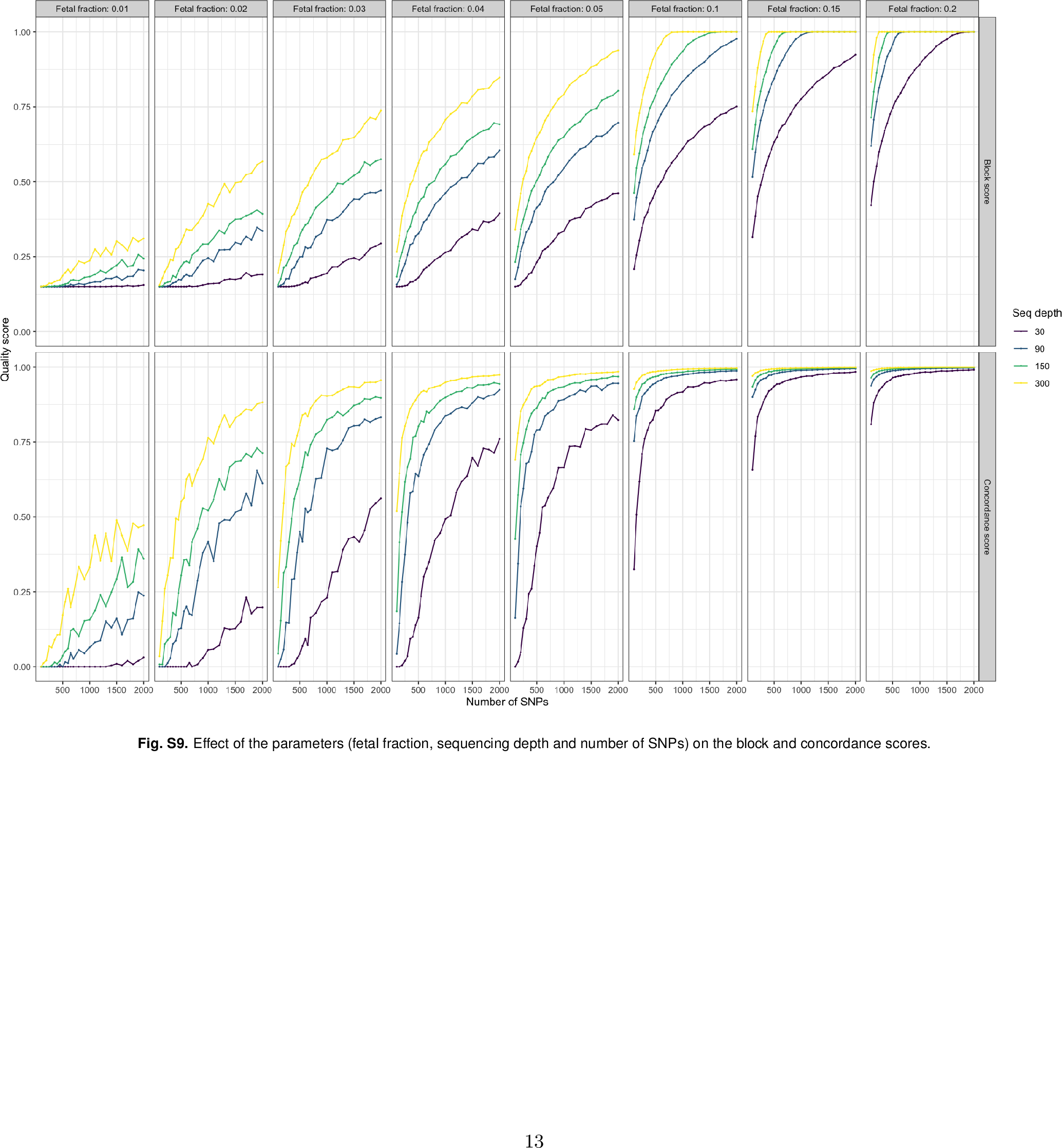

Supplement: S9 Fig — (TIF) [file pone.0280976.s009.tif]
