# Supplementary material for: Non-invasive prenatal diagnosis of single gene disorders with enhanced relative haplotype dosage analysis for diagnostic implementation
Source: PLoS One. 2023 Apr 24;18(4):e0280976. doi: 10.1371/journal.pone.0280976 (PMC10124834; doi:10.1371/journal.pone.0280976)
Supplement: S1 Table — (PDF) [file pone.0280976.s010.pdf]

Supplemental Data for

Non-Invasive Prenatal Diagnosis of Single Gene Disorders with enhanced Relative Haplotype Dosage Analysis for diagnosis implementation

Mathilde Pacault, Camille Verebi, Magali Champion, Lucie Orhant, Alexandre Perrier, Emmanuelle Girodon, France Leturcq, Dominique Vidaud, Claude Férec, Thierry Bienvenu, Romain Daveau, Juliette Nectoux

Supplementary Table S1

Table S1. SNP categorization for autosomal inheritance, taking into account the proband's relationship to the pregnant couple and his status towards the familial pathogenic variants

Proband = Affected child

SNP type	SNP Subtype	Maternal Genotype	Paternal Genotype	Proband Genotype	Maternal		Paternal		Information from genotype	Result interpretation	SPRT analysis	
					Risk HapI	Non at risk HapII	Risk HapIII	Non at risk HapIV			q_0	q_1
1	A	AA	BB	AB	ND	ND	ND	ND	Detection of paternal contribution but not specific allele	Fetal Fraction	ND	ND
1	B	BB	AA	AB	ND	ND	ND	ND	Detection of paternal contribution but not specific allele	Fetal Fraction	ND	ND
2	A	AA	AA	AA	ND	ND	ND	ND	Sequencing error rate	Quality Control	ND	ND
2	B	BB	BB	BB	ND	ND	ND	ND	Sequencing error rate	Quality Control	ND	ND
3	A	AA	**A***B	A**A***	ND	ND	**A***	B	Detection of paternal-specific allele	If B detected: fetal haplotype HapIV<br>if not: fetal haplotype HapIII	ND	ND
3	B	AA	A**B***	A**B***	ND	ND	**B***	A	Detection of paternal-specific allele	If B detected: fetal haplotype HapIII<br>if not: fetal haplotype HapIV	ND	ND
3	C	BB	**A***B	**A***B	ND	ND	**A***	B	Detection of paternal-specific allele	If A detected: fetal haplotype HapIII<br>if not: fetal haplotype HapIV	ND	ND
3	D	BB	A**B***	B**B***	ND	ND	**B***	A	Detection of paternal-specific allele	If A detected: fetal haplotype HapIV<br>if not: fetal haplotype HapIII	ND	ND
4α	A	**A***B	AA	A**A***	**A***	B	ND	ND	Detection of maternal-specific allele	If A > B: fetal haplotype HapI<br>if A = B: fetal haplotype HapII	0.5	(1+f)/2
4α	B	A**B***	BB	B**B***	**B***	A	ND	ND	Detection of maternal-specific allele	If B > A: fetal haplotype HapI<br>if A = B: fetal haplotype HapII	0.5	(1+f)/2
4β	A	**A***B	BB	**A***B	**A***	B	ND	ND	Detection of maternal-specific allele	If A = B: fetal haplotype HapI<br>if B > A: fetal haplotype HapII	(1-f)/2	0.5
4β	B	A**B***	AA	A**B***	**B***	A	ND	ND	Detection of maternal-specific allele	If A = B: fetal haplotype HapI<br>if A > B: fetal haplotype HapII	(1-f)/2	0.5
WARNING_2	A	AB	AB	AA	ND	ND	ND	ND	Genotype Combination Non Informative	Genotype Combination Non Informative	ND	ND
WARNING_2	B	AB	AB	AB	ND	ND	ND	ND	Genotype Combination Non Informative	Genotype Combination Non Informative	ND	ND
WARNING_2	C	AB	AB	BB	ND	ND	ND	ND	Genotype Combination Non Informative	Genotype Combination Non Informative	ND	ND
WARNING_1	A	AA	BB	AA	ND	ND	ND	ND	Genotype Combination Impossible	Genotype Combination Impossible	ND	ND
WARNING_1	B	AA	BB	BB	ND	ND	ND	ND	Genotype Combination Impossible	Genotype Combination Impossible	ND	ND
WARNING_1	C	BB	AA	AA	ND	ND	ND	ND	Genotype Combination Impossible	Genotype Combination Impossible	ND	ND
WARNING_1	D	BB	AA	BB	ND	ND	ND	ND	Genotype Combination Impossible	Genotype Combination Impossible	ND	ND
WARNING_1	E	AA	AA	AB	ND	ND	ND	ND	Genotype Combination Impossible	Genotype Combination Impossible	ND	ND
WARNING_1	F	AA	AA	BB	ND	ND	ND	ND	Genotype Combination Impossible	Genotype Combination Impossible	ND	ND
WARNING_1	G	BB	BB	AA	ND	ND	ND	ND	Genotype Combination Impossible	Genotype Combination Impossible	ND	ND
WARNING_1	H	BB	BB	AB	ND	ND	ND	ND	Genotype Combination Impossible	Genotype Combination Impossible	ND	ND
WARNING_1	I	AA	AB	BB	ND	ND	ND	ND	Genotype Combination Impossible	Genotype Combination Impossible	ND	ND
WARNING_1	J	BB	AB	AA	ND	ND	ND	ND	Genotype Combination Impossible	Genotype Combination Impossible	ND	ND
WARNING_1	K	AB	BB	AA	ND	ND	ND	ND	Genotype Combination Impossible	Genotype Combination Impossible	ND	ND
WARNING_1	L	AB	AA	BB	ND	ND	ND	ND	Genotype Combination Impossible	Genotype Combination Impossible	ND	ND

Proband = Unaffected/Non Carrier Child

SNP type	SNP Subtype	Maternal Genotype	Paternal Genotype	Proband Genotype	Maternal Risk HapI	Maternal Non at risk HapII	Paternal Risk HapIII	Paternal Non at risk HapIV	Information from genotype	Result interpretation	SPRT analysis q_0	SPRT analysis q_1
1	A	AA	BB	AB	ND	ND	ND	ND	Detection of paternal contribution but not specific allele	Fetal Fraction	ND	ND
1	B	BB	AA	AB	ND	ND	ND	ND	Detection of paternal contribution but not specific allele	Fetal Fraction	ND	ND
2	A	AA	AA	AA	ND	ND	ND	ND	Sequencing error rate	Quality Control	ND	ND
2	B	BB	BB	BB	ND	ND	ND	ND	Sequencing error rate	Quality Control	ND	ND
3	E	AA	**A***B	AB	ND	ND	**A***	B	Detection of paternal-specific allele	If B detected: fetal haplotype HapIV if not: fetal haplotype HapIII	ND	ND
3	F	AA	A**B***	AA	ND	ND	**B***	A	Detection of paternal-specific allele	If B detected: fetal haplotype HapIII if not: fetal haplotype HapIV	ND	ND
3	G	BB	**A***B	BB	ND	ND	**A***	B	Detection of paternal-specific allele	If A detected: fetal haplotype HapIII if not: fetal haplotype HapIV	ND	ND
3	H	BB	A**B***	AB	ND	ND	**B***	A	Detection of paternal-specific allele	If A detected: fetal haplotype HapIV if not: fetal haplotype HapIII	ND	ND
4α	C	**A***B	AA	AB	**A***	B	ND	ND	Detection of maternal-specific allele	If A > B: fetal haplotype HapI if A = B: fetal haplotype HapII	0.5	(1+f)/2
4α	D	A**B***	BB	AB	**B***	A	ND	ND	Detection of maternal-specific allele	If B > A: fetal haplotype HapI if A = B: fetal haplotype HapII	0.5	(1+f)/2
4β	C	**A***B	BB	BB	**A***	B	ND	ND	Detection of maternal-specific allele	If A = B: fetal haplotype HapI if B > A: fetal haplotype HapII	(1-f)/2	0.5
4β	D	A**B***	AA	AA	**B***	A	ND	ND	Detection of maternal-specific allele	If A = B: fetal haplotype HapI if A > B: fetal haplotype HapII	(1-f)/2	0.5
WARNING_2	A	AB	AB	AA	ND	ND	ND	ND	Genotype Combination Non Informative	Genotype Combination Non Informative	ND	ND
WARNING_2	B	AB	AB	AB	ND	ND	ND	ND	Genotype Combination Non Informative	Genotype Combination Non Informative	ND	ND
WARNING_2	C	AB	AB	BB	ND	ND	ND	ND	Genotype Combination Non Informative	Genotype Combination Non Informative	ND	ND
WARNING_1	A	AA	BB	AA	ND	ND	ND	ND	Genotype Combination Impossible	Genotype Combination Impossible	ND	ND
WARNING_1	B	AA	BB	BB	ND	ND	ND	ND	Genotype Combination Impossible	Genotype Combination Impossible	ND	ND
WARNING_1	C	BB	AA	AA	ND	ND	ND	ND	Genotype Combination Impossible	Genotype Combination Impossible	ND	ND
WARNING_1	D	BB	AA	BB	ND	ND	ND	ND	Genotype Combination Impossible	Genotype Combination Impossible	ND	ND
WARNING_1	E	AA	AA	AB	ND	ND	ND	ND	Genotype Combination Impossible	Genotype Combination Impossible	ND	ND
WARNING_1	F	AA	AA	BB	ND	ND	ND	ND	Genotype Combination Impossible	Genotype Combination Impossible	ND	ND
WARNING_1	G	BB	BB	AA	ND	ND	ND	ND	Genotype Combination Impossible	Genotype Combination Impossible	ND	ND
WARNING_1	H	BB	BB	AB	ND	ND	ND	ND	Genotype Combination Impossible	Genotype Combination Impossible	ND	ND
WARNING_1	I	AA	AB	BB	ND	ND	ND	ND	Genotype Combination Impossible	Genotype Combination Impossible	ND	ND
WARNING_1	J	BB	AB	AA	ND	ND	ND	ND	Genotype Combination Impossible	Genotype Combination Impossible	ND	ND
WARNING_1	K	AB	BB	AA	ND	ND	ND	ND	Genotype Combination Impossible	Genotype Combination Impossible	ND	ND
WARNING_1	L	AB	AA	BB	ND	ND	ND	ND	Genotype Combination Impossible	Genotype Combination Impossible	ND	ND

Proband = Affected Close Relative

SNP type	SNP Subtype	Maternal Genotype	Paternal Genotype	Proband Genotype	Maternal Risk HapI	Maternal Non at risk HapII	Paternal Risk HapIII	Paternal Non at risk HapIV	Information from genotype	Result interpretation	SPRT analysis q_0	SPRT analysis q_1
1	A	AA	BB	AB	ND	ND	ND	ND	Detection of paternal contribution but not specific allele	Fetal Fraction	ND	ND
1	B	BB	AA	AB	ND	ND	ND	ND	Detection of paternal contribution but not specific allele	Fetal Fraction	ND	ND
1	C	AA	BB	AA	ND	ND	ND	ND	Detection of paternal contribution but not specific allele	Fetal Fraction	ND	ND
1	D	BB	AA	AA	ND	ND	ND	ND	Detection of paternal contribution but not specific allele	Fetal Fraction	ND	ND
1	E	AA	BB	BB	ND	ND	ND	ND	Detection of paternal contribution but not specific allele	Fetal Fraction	ND	ND
1	F	BB	AA	BB	ND	ND	ND	ND	Detection of paternal contribution but not specific allele	Fetal Fraction	ND	ND
2	A	AA	AA	AA	ND	ND	ND	ND	Sequencing error rate	Quality Control	ND	ND
2	B	BB	BB	BB	ND	ND	ND	ND	Sequencing error rate	Quality Control	ND	ND
2	C	AA	AA	AB	ND	ND	ND	ND	Sequencing error rate	Quality Control	ND	ND
2	D	AA	AA	BB	ND	ND	ND	ND	Sequencing error rate	Quality Control	ND	ND
2	E	BB	BB	AA	ND	ND	ND	ND	Sequencing error rate	Quality Control	ND	ND
2	F	BB	BB	AB	ND	ND	ND	ND	Sequencing error rate	Quality Control	ND	ND
3	A	AA	**A***B	A**A***	ND	ND	**A***	B	Detection of paternal-specific allele	If B detected: fetal haplotype HapIV if not: fetal haplotype HapIII	ND	ND
3	D	BB	A**B***	B**B***	ND	ND	**B***	A	Detection of paternal-specific allele	If A detected: fetal haplotype HapIV if not: fetal haplotype HapIII	ND	ND
3	I	BB	**A***B	A**A***	ND	ND	**A***	B	Detection of paternal-specific allele	If A detected: fetal haplotype HapIII if not: fetal haplotype HapIV	ND	ND
3	J	AA	A**B***	B**B***	ND	ND	**B***	A	Detection of paternal-specific allele	If B detected: fetal haplotype HapIII if not: fetal haplotype HapIV	ND	ND
4α	A	**A***B	AA	A**A***	**A***	B	ND	ND	Detection of maternal-specific allele	If A>B: fetal haplotype HapI if A = B: fetal haplotype HapII	0.5	(1+f)/2
4α	B	A**B***	BB	B**B***	**B***	A	ND	ND	Detection of maternal-specific allele	If B>A: fetal haplotype HapI if A = B: fetal haplotype HapII	0.5	(1+f)/2
4β	E	**A***B	BB	A**A***	**A***	B	ND	ND	Detection of maternal-specific allele	If A=B: fetal haplotype HapI if B = A: fetal haplotype HapII	(1-f)/2	0.5
4β	F	A**B***	AA	B**B***	**B***	A	ND	ND	Detection of maternal-specific allele	If A=B: fetal haplotype HapI if A > B: fetal haplotype HapII	(1-f)/2	0.5
WARNING_2	A	AB	AB	AA	ND	ND	ND	ND	Genotype Combination Non Informative	Genotype Combination Non Informative	ND	ND
WARNING_2	B	AB	AB	AB	ND	ND	ND	ND	Genotype Combination Non Informative	Genotype Combination Non Informative	ND	ND
WARNING_2	C	AB	AB	BB	ND	ND	ND	ND	Genotype Combination Non Informative	Genotype Combination Non Informative	ND	ND
WARNING_2	D	AA	AB	AB	ND	ND	ND	ND	Genotype Combination Non Informative	Genotype Combination Non Informative	ND	ND
WARNING_2	E	BB	AB	AB	ND	ND	ND	ND	Genotype Combination Non Informative	Genotype Combination Non Informative	ND	ND
WARNING_2	F	AB	AA	AB	ND	ND	ND	ND	Genotype Combination Non Informative	Genotype Combination Non Informative	ND	ND
WARNING_2	G	AB	BB	AB	ND	ND	ND	ND	Genotype Combination Non Informative	Genotype Combination Non Informative	ND	ND

Proband = Unaffected/Non Carrier Close Relative

SNP type	SNP Subtype	Maternal Genotype	Paternal Genotype	Proband Genotype	Maternal Risk HapI	Maternal Non at risk HapII	Paternal Risk HapIII	Paternal Non at risk HapIV	Information from genotype	Result interpretation	SPRT analysis q_0	SPRT analysis q_1
1	A	AA	BB	AB	ND	ND	ND	ND	Detection of paternal contribution but not specific allele	Fetal Fraction	ND	ND
1	B	BB	AA	AB	ND	ND	ND	ND	Detection of paternal contribution but not specific allele	Fetal Fraction	ND	ND
1	C	AA	BB	AA	ND	ND	ND	ND	Detection of paternal contribution but not specific allele	Fetal Fraction	ND	ND
1	D	BB	AA	AA	ND	ND	ND	ND	Detection of paternal contribution but not specific allele	Fetal Fraction	ND	ND
1	E	AA	BB	BB	ND	ND	ND	ND	Detection of paternal contribution but not specific allele	Fetal Fraction	ND	ND
1	F	BB	AA	BB	ND	ND	ND	ND	Detection of paternal contribution but not specific allele	Fetal Fraction	ND	ND
2	A	AA	AA	AA	ND	ND	ND	ND	Sequencing error rate	Quality Control	ND	ND
2	B	BB	BB	BB	ND	ND	ND	ND	Sequencing error rate	Quality Control	ND	ND
2	C	AA	AA	AB	ND	ND	ND	ND	Sequencing error rate	Quality Control	ND	ND
2	D	AA	AA	BB	ND	ND	ND	ND	Sequencing error rate	Quality Control	ND	ND
2	E	BB	BB	AA	ND	ND	ND	ND	Sequencing error rate	Quality Control	ND	ND
2	F	BB	BB	AB	ND	ND	ND	ND	Sequencing error rate	Quality Control	ND	ND
3	E	AA	**A***B	AA	ND	ND	**A***	B	Detection of paternal-specific allele	If B detected: fetal haplotype HapIII<br>if not: fetal haplotype HapIV	ND	ND
3	H	BB	A**B***	BB	ND	ND	**B***	A	Detection of paternal-specific allele	If A detected: fetal haplotype HapIII<br>if not: fetal haplotype HapIV	ND	ND
3	K	BB	**A***B	AA	ND	ND	**A***	B	Detection of paternal-specific allele	If A detected: fetal haplotype HapIV<br>if not: fetal haplotype HapIII	ND	ND
3	L	AA	A**B***	BB	ND	ND	**B***	A	Detection of paternal-specific allele	If B detected: fetal haplotype HapIV<br>if not: fetal haplotype HapIII	ND	ND
4α	E	A**B***	BB	AA	**B***	A	ND	ND	Detection of maternal-specific allele	If B>A: fetal haplotype HapI<br>if A=B: fetal haplotype HapII	0.5	(1+f)/2
4α	F	**A***B	AA	BB	**A***	B	ND	ND	Detection of maternal-specific allele	If A>B: fetal haplotype HapI<br>if A=B: fetal haplotype HapII	0.5	(1+f)/2
4β	C	**A***B	BB	BB	**A***	B	ND	ND	Detection of maternal-specific allele	If A=B: fetal haplotype HapI<br>if B>A: fetal haplotype HapII	(1-f)/2	0.5
4β	D	A**B***	AA	AA	**B***	A	ND	ND	Detection of maternal-specific allele	If A=B: fetal haplotype HapI<br>if A>B: fetal haplotype HapII	(1-f)/2	0.5
WARNING_2	A	AB	AB	AA	ND	ND	ND	ND	Genotype Combination Non Informative	Genotype Combination Non Informative	ND	ND
WARNING_2	B	AB	AB	AB	ND	ND	ND	ND	Genotype Combination Non Informative	Genotype Combination Non Informative	ND	ND
WARNING_2	C	AB	AB	BB	ND	ND	ND	ND	Genotype Combination Non Informative	Genotype Combination Non Informative	ND	ND
WARNING_2	D	AA	AB	AB	ND	ND	ND	ND	Genotype Combination Non Informative	Genotype Combination Non Informative	ND	ND
WARNING_2	E	BB	AB	AB	ND	ND	ND	ND	Genotype Combination Non Informative	Genotype Combination Non Informative	ND	ND
WARNING_2	F	AB	AA	AB	ND	ND	ND	ND	Genotype Combination Non Informative	Genotype Combination Non Informative	ND	ND
WARNING_2	G	AB	BB	AB	ND	ND	ND	ND	Genotype Combination Non Informative	Genotype Combination Non Informative	ND	ND
