# Supplementary material for: Non-invasive prenatal diagnosis of single gene disorders with enhanced relative haplotype dosage analysis for diagnostic implementation
Source: PLoS One. 2023 Apr 24;18(4):e0280976. doi: 10.1371/journal.pone.0280976 (PMC10124834; doi:10.1371/journal.pone.0280976)
Supplement: S2 Table — (PDF) [file pone.0280976.s011.pdf]

Supplemental Data for

Non-Invasive Prenatal Diagnosis of Single Gene Disorders with enhanced Relative Haplotype Dosage Analysis for diagnosis implementation

Mathilde Pacault, Camille Verebi, Magali Champion, Lucie Orhant, Alexandre Perrier, Emmanuelle Girodon, France Leturcq, Dominique Vidaud, Claude Férec, Thierry Bienvenu, Romain Daveau, Juliette Nectoux

Supplementary Table S2

Table S2: SNP categorization for X-linked inheritance, taking into account the fetal gender, the proband's gender, his relationship to the pregnant couple and his status towards the familial pathogenic variants

SNP type	SNP Subtype	Maternal Genotype	Paternal Genotype	Proband Genotype	Maternal at-risk HapI	Maternal non-at-risk HapII	Information from genotype	Result interpretation	SPRT analysis q_0	SPRT analysis q_1
**Clinical setting**		**"Male Fetus" AND "Proband = Carrier/ Affected Child, Female"**								
2	A	AA	A	AA	NA	NA	Sequencing error rate	Quality Control	NA	NA
2	B	BB	B	BB	NA	NA	Sequencing error rate	Quality Control	NA	NA
4X	A	**A***B	A	A**A***	**A***	B	Detection of maternal-specific allele	If A >B : fetal haplotype HapI<br>If B >A : fetal haplotype HapII	(1-f)/(2-f)	1/(2-f)
4X	B	A**B***	B	B**B***	**B***	A	Detection of maternal-specific allele	If B >A : fetal haplotype HapI<br>If A >B : fetal haplotype HapII	(1-f)/(2-f)	1/(2-f)
4X	C	**A***B	B	**A***B	**A***	B	Detection of maternal-specific allele	If B >A : fetal haplotype HapII<br>If A >B : fetal haplotype HapI	(1-f)/(2-f)	1/(2-f)
4X	D	A**B***	A	A**B***	**B***	A	Detection of maternal-specific allele	If A >B : fetal haplotype HapII<br>If B >A : fetal haplotype HapI	(1-f)/(2-f)	1/(2-f)
WARNING_1	NA	AA	A	AB	NA	NA	Genotype Combination Impossible		NA	NA
WARNING_1	NA	AA	A	BB	NA	NA	Genotype Combination Impossible		NA	NA
WARNING_1	NA	AB	A	BB	NA	NA	Genotype Combination Impossible		NA	NA
WARNING_1	NA	BB	A	AA	NA	NA	Genotype Combination Impossible		NA	NA
WARNING_1	NA	BB	A	BB	NA	NA	Genotype Combination Impossible		NA	NA
WARNING_1	NA	AA	B	AA	NA	NA	Genotype Combination Impossible		NA	NA
WARNING_1	NA	AA	B	BB	NA	NA	Genotype Combination Impossible		NA	NA
WARNING_1	NA	AB	B	AA	NA	NA	Genotype Combination Impossible		NA	NA
WARNING_1	NA	BB	B	AA	NA	NA	Genotype Combination Impossible		NA	NA
WARNING_1	NA	BB	B	AB	NA	NA	Genotype Combination Impossible		NA	NA
WARNING_2	NA	AA	B	AB	NA	NA	Genotype Combination Uninformative		NA	NA
WARNING_2	NA	BB	A	AB	NA	NA	Genotype Combination Uninformative		NA	NA
**Clinical setting**		**"Male Fetus" AND "Proband = Affected Child, Male"**								
2	C	AA	A	A	NA	NA	Sequencing error rate	Quality Control	NA	NA
2	D	BB	B	B	NA	NA	Sequencing error rate	Quality Control	NA	NA
4X	E	**A***B	A	**A***	**A***	B	Detection of maternal-specific allele	If A >B : fetal haplotype HapI<br>If B >A : fetal haplotype HapII	(1-f)/(2-f)	1/(2-f)
4X	F	A**B***	B	**B***	**B***	A	Detection of maternal-specific allele	If B >A : fetal haplotype HapI<br>If A >B : fetal haplotype HapII	(1-f)/(2-f)	1/(2-f)
4X	G	**A***B	B	**A***	**A***	B	Detection of maternal-specific allele	If B >A : fetal haplotype HapII<br>If A >B : fetal haplotype HapI	(1-f)/(2-f)	1/(2-f)
4X	H	A**B***	A	**B***	**B***	A	Detection of maternal-specific allele	If A >B : fetal haplotype HapII<br>If B >A : fetal haplotype HapI	(1-f)/(2-f)	1/(2-f)
WARNING_1	NA	AA	A	B	NA	NA	Genotype Combination Impossible		NA	NA
WARNING_1	NA	BB	A	A	NA	NA	Genotype Combination Impossible		NA	NA
WARNING_1	NA	AA	B	B	NA	NA	Genotype Combination Impossible		NA	NA
WARNING_1	NA	BB	B	A	NA	NA	Genotype Combination Impossible		NA	NA
WARNING_2	NA	AA	B	A	NA	NA	Genotype Combination Uninformative		NA	NA
WARNING_2	NA	BB	A	B	NA	NA	Genotype Combination Uninformative		NA	NA

SNP type	SNP Subtype	Maternal Genotype	Paternal Genotype	Proband Genotype	Maternal at-risk HapI	Maternal non-at-risk HapII	Information from genotype	Result interpretation	SPRT analysis q_0	q_1
**Clinical setting**		**"Male Fetus" AND "Proband = Non Carrier/ Non affected Child, Female"**								
2	A	AA	A	AA	NA	NA	Sequencing error rate	Quality Control	NA	NA
2	B	BB	B	BB	NA	NA	Sequencing error rate	Quality Control	NA	NA
4X	I	**A***B	A	AB	**A***	B	Detection of maternal-specific allele	If A >B : fetal haplotype HapI<br>If B >A : fetal haplotype HapII	(1-f)/(2-f)	1/(2-f)
4X	J	A**B***	B	AB	**B***	A	Detection of maternal-specific allele	If B >A : fetal haplotype HapI<br>If A >B : fetal haplotype HapII	(1-f)/(2-f)	1/(2-f)
4X	K	**A***B	B	BB	**A***	B	Detection of maternal-specific allele	If B >A : fetal haplotype HapII<br>If A >B : fetal haplotype HapI	(1-f)/(2-f)	1/(2-f)
4X	L	A**B***	A	AA	**B***	A	Detection of maternal-specific allele	If A >B : fetal haplotype HapII<br>If B >A : fetal haplotype HapI	(1-f)/(2-f)	1/(2-f)
WARNING_1	NA	AA	A	AB	NA	NA	Genotype Combination Impossible		NA	NA
WARNING_1	NA	AA	A	BB	NA	NA	Genotype Combination Impossible		NA	NA
WARNING_1	NA	AB	A	BB	NA	NA	Genotype Combination Impossible		NA	NA
WARNING_1	NA	BB	A	AA	NA	NA	Genotype Combination Impossible		NA	NA
WARNING_1	NA	BB	A	BB	NA	NA	Genotype Combination Impossible		NA	NA
WARNING_1	NA	AA	B	AA	NA	NA	Genotype Combination Impossible		NA	NA
WARNING_1	NA	AA	B	BB	NA	NA	Genotype Combination Impossible		NA	NA
WARNING_1	NA	AB	B	AA	NA	NA	Genotype Combination Impossible		NA	NA
WARNING_1	NA	BB	B	AA	NA	NA	Genotype Combination Impossible		NA	NA
WARNING_1	NA	BB	B	AB	NA	NA	Genotype Combination Impossible		NA	NA
WARNING_2	NA	AA	B	AB	NA	NA	Genotype Combination Uninformative		NA	NA
WARNING_2	NA	BB	A	AB	NA	NA	Genotype Combination Uninformative		NA	NA
**Clinical setting**		**"Male Fetus" AND "Proband = Unaffected Child, Male"**								
2	C	AA	A	A	NA	NA	Sequencing error rate	Quality Control	NA	NA
2	D	BB	B	B	NA	NA	Sequencing error rate	Quality Control	NA	NA
4X	M	**A***B	A	B	**A***	B	Detection of maternal-specific allele	If A >B : fetal haplotype HapI<br>If B >A : fetal haplotype HapII	(1-f)/(2-f)	1/(2-f)
4X	N	A**B***	B	A	**B***	A	Detection of maternal-specific allele	If B >A : fetal haplotype HapI<br>If A >B : fetal haplotype HapII	(1-f)/(2-f)	1/(2-f)
4X	O	**A***B	B	B	**A***	B	Detection of maternal-specific allele	If B >A : fetal haplotype HapII<br>If A >B : fetal haplotype HapI	(1-f)/(2-f)	1/(2-f)
4X	P	A**B***	A	A	**B***	A	Detection of maternal-specific allele	If A >B : fetal haplotype HapII<br>If B >A : fetal haplotype HapI	(1-f)/(2-f)	1/(2-f)
WARNING_1	NA	AA	A	B	NA	NA	Genotype Combination Impossible		NA	NA
WARNING_1	NA	BB	A	A	NA	NA	Genotype Combination Impossible		NA	NA
WARNING_1	NA	AA	B	B	NA	NA	Genotype Combination Impossible		NA	NA
WARNING_1	NA	BB	B	A	NA	NA	Genotype Combination Impossible		NA	NA
WARNING_2	NA	AA	B	A	NA	NA	Genotype Combination Uninformative		NA	NA
WARNING_2	NA	BB	A	B	NA	NA	Genotype Combination Uninformative		NA	NA

	SNP type	SNP Subtype	Maternal Genotype	Paternal Genotype	Proband Genotype	Maternal at-risk HapI	Maternal non-at-risk HapII	Information from genotype	Result interpretation	SPRT analysis q_0	q_1
**Clinical setting**			**"Female Fetus" AND "Proband = Carrier/ Affected Child, Female"**								
	1	A	AA	B	AB	NA	NA	Detection of paternal contribution	Fetal Fraction	NA	NA
	1	B	BB	A	AB	NA	NA	Detection of paternal contribution	Fetal Fraction	NA	NA
	2	A	AA	A	AA	NA	NA	Sequencing error rate	Quality Control	NA	NA
	2	B	BB	B	BB	NA	NA	Sequencing error rate	Quality Control	NA	NA
	4X	AA	**A***B	A	A**A***	**A***	B	Detection of maternal-specific allele	If A >B : fetal haplotype HapI<br>if A = B : fetal haplotype HapII	0.5	(1+f)/2
	4X	BB	A**B***	B	B**B***	**B***	A	Detection of maternal-specific allele	If B >A : fetal haplotype HapI<br>if A = B : fetal haplotype HapII	0.5	(1+f)/2
	4X	CC	**A***B	B	**A***B	**A***	B	Detection of maternal-specific allele	If B >A : fetal haplotype HapII<br>if A = B : fetal haplotype HapI	(1-f)/2	0.5
	4X	DD	A**B***	A	A**B***	**B***	A	Detection of maternal-specific allele	If A >B : fetal haplotype HapII<br>if A = B : fetal haplotype HapI	(1-f)/2	0.5
	WARNING_1	NA	AA	A	AB	NA	NA	Genotype Combination Impossible		NA	NA
	WARNING_1	NA	AA	A	BB	NA	NA	Genotype Combination Impossible		NA	NA
	WARNING_1	NA	AB	A	BB	NA	NA	Genotype Combination Impossible		NA	NA
	WARNING_1	NA	BB	A	AA	NA	NA	Genotype Combination Impossible		NA	NA
	WARNING_1	NA	BB	A	BB	NA	NA	Genotype Combination Impossible		NA	NA
	WARNING_1	NA	AA	B	AA	NA	NA	Genotype Combination Impossible		NA	NA
	WARNING_1	NA	AA	B	BB	NA	NA	Genotype Combination Impossible		NA	NA
	WARNING_1	NA	AB	B	AA	NA	NA	Genotype Combination Impossible		NA	NA
	WARNING_1	NA	BB	B	AA	NA	NA	Genotype Combination Impossible		NA	NA
	WARNING_1	NA	BB	B	AB	NA	NA	Genotype Combination Impossible		NA	NA
**Clinical setting**			**"Female Fetus" AND "Proband = Affected Child, Male"**								
	1	C	AA	B	A	NA	NA	Detection of paternal contribution	Fetal Fraction	NA	NA
	1	D	BB	A	B	NA	NA	Detection of paternal contribution	Fetal Fraction	NA	NA
	2	C	AA	A	A	NA	NA	Sequencing error rate	Quality Control	NA	NA
	2	D	BB	B	B	NA	NA	Sequencing error rate	Quality Control	NA	NA
	4X	EE	**A***B	A	**A***	**A***	B	Detection of maternal-specific allele	If A >B : fetal haplotype HapI<br>if A = B : fetal haplotype HapII	0.5	(1+f)/2
	4X	FF	A**B***	B	**B***	**B***	A	Detection of maternal-specific allele	If B >A : fetal haplotype HapI<br>if A = B : fetal haplotype HapII	0.5	(1+f)/2
	4X	GG	**A***B	B	**A***	**A***	B	Detection of maternal-specific allele	If B >A : fetal haplotype HapII<br>if A = B : fetal haplotype HapI	(1-f)/2	0.5
	4X	HH	A**B***	A	**B***	**B***	A	Detection of maternal-specific allele	If A >B : fetal haplotype HapII<br>if A = B : fetal haplotype HapI	(1-f)/2	0.5
	WARNING_1	NA	AA	A	B	NA	NA	Genotype Combination Impossible		NA	NA
	WARNING_1	NA	BB	A	A	NA	NA	Genotype Combination Impossible		NA	NA
	WARNING_1	NA	AA	B	B	NA	NA	Genotype Combination Impossible		NA	NA
	WARNING_1	NA	BB	B	A	NA	NA	Genotype Combination Impossible		NA	NA

SNP type	SNP Subtype	Maternal Genotype	Paternal Genotype	Proband Genotype	Maternal at-risk HapI	Maternal non-at-risk HapII	Information from genotype	Result interpretation	SPRT analysis q_0	q_1
**Clinical setting**		**"Female Fetus" AND "Proband = Non Carrier/ Non affected Child, Female"**								
1	A	AA	B	AB	NA	NA	Detection of paternal contribution	Fetal Fraction	NA	NA
1	B	BB	A	AB	NA	NA	Detection of paternal contribution	Fetal Fraction	NA	NA
2	A	AA	A	AA	NA	NA	Sequencing error rate	Quality Control	NA	NA
2	B	BB	B	BB	NA	NA	Sequencing error rate	Quality Control	NA	NA
4X	II	**A***B	A	AB	**A***	B	Detection of maternal-specific allele	If A >B : fetal haplotype HapI<br>if A = B : fetal haplotype HapII	0.5	(1+f)/2
4X	JJ	A**B***	B	AB	**B***	A	Detection of maternal-specific allele	If B >A : fetal haplotype HapI<br>if A = B : fetal haplotype HapII	0.5	(1+f)/2
4X	KK	**A***B	B	BB	**A***	B	Detection of maternal-specific allele	If B >A : fetal haplotype HapII<br>if A = B : fetal haplotype HapI	(1-f)/2	0.5
4X	LL	A**B***	A	AA	**B***	A	Detection of maternal-specific allele	If A >B : fetal haplotype HapII<br>if A = B : fetal haplotype HapI	(1-f)/2	0.5
WARNING_1	NA	AA	A	AB	NA	NA	Genotype Combination Impossible		NA	NA
WARNING_1	NA	AA	A	BB	NA	NA	Genotype Combination Impossible		NA	NA
WARNING_1	NA	AB	A	BB	NA	NA	Genotype Combination Impossible		NA	NA
WARNING_1	NA	BB	A	AA	NA	NA	Genotype Combination Impossible		NA	NA
WARNING_1	NA	BB	A	BB	NA	NA	Genotype Combination Impossible		NA	NA
WARNING_1	NA	AA	B	AA	NA	NA	Genotype Combination Impossible		NA	NA
WARNING_1	NA	AA	B	BB	NA	NA	Genotype Combination Impossible		NA	NA
WARNING_1	NA	AB	B	AA	NA	NA	Genotype Combination Impossible		NA	NA
WARNING_1	NA	BB	B	AA	NA	NA	Genotype Combination Impossible		NA	NA
WARNING_1	NA	BB	B	AB	NA	NA	Genotype Combination Impossible		NA	NA
**Clinical setting**		**"Female Fetus" AND "Proband = Unaffected Child, Male"**								
1	C	AA	B	A	NA	NA	Detection of paternal contribution	Fetal Fraction	NA	NA
1	D	BB	A	B	NA	NA	Detection of paternal contribution	Fetal Fraction	NA	NA
2	C	AA	A	A	NA	NA	Sequencing error rate	Quality Control	NA	NA
2	D	BB	B	B	NA	NA	Sequencing error rate	Quality Control	NA	NA
4X	MM	**A***B	A	B	**A***	B	Detection of maternal-specific allele	If A >B : fetal haplotype HapI<br>if A = B : fetal haplotype HapII	0.5	(1+f)/2
4X	NN	A**B***	B	A	**B***	A	Detection of maternal-specific allele	If B >A : fetal haplotype HapI<br>if A = B : fetal haplotype HapII	0.5	(1+f)/2
4X	OO	**A***B	B	B	**A***	B	Detection of maternal-specific allele	If B >A : fetal haplotype HapII<br>if A = B : fetal haplotype HapI	(1-f)/2	0.5
4X	PP	A**B***	A	A	**B***	A	Detection of maternal-specific allele	If A >B : fetal haplotype HapII<br>if A = B : fetal haplotype HapI	(1-f)/2	0.5
WARNING_1	NA	AA	A	B	NA	NA	Genotype Combination Impossible		NA	NA
WARNING_1	NA	BB	A	A	NA	NA	Genotype Combination Impossible		NA	NA
WARNING_1	NA	AA	B	B	NA	NA	Genotype Combination Impossible		NA	NA
WARNING_1	NA	BB	B	A	NA	NA	Genotype Combination Impossible		NA	NA

SNP type	SNP Subtype	Maternal Genotype	Paternal Genotype	Proband Genotype	Maternal at-risk HapI	Maternal non-at-risk HapII	Information from genotype	Result interpretation	SPRT analysis q_0	q_1
**Clinical setting**		**"Male Fetus" AND "Proband = Maternal Carrier/ Affected Close Relative, Female"**								
2	A	AA	A	AA	NA	NA	Sequencing error rate	Quality Control	NA	NA
2	B	BB	B	BB	NA	NA	Sequencing error rate	Quality Control	NA	NA
2	E	AA	A	AB	NA	NA	Sequencing error rate	Quality Control	NA	NA
2	F	BB	B	AB	NA	NA	Sequencing error rate	Quality Control	NA	NA
4X	A	A*B	A	AA*	A*	B	Detection of maternal-specific allele	If A >B : fetal haplotype HapI<br>if B >A : fetal haplotype HapII	(1-f)/(2-f)	1/(2-f)
4X	B	AB*	B	BB*	B*	A	Detection of maternal-specific allele	If B >A : fetal haplotype HapI<br>if A >B : fetal haplotype HapII	(1-f)/(2-f)	1/(2-f)
4X	Q	AB*	A	BB*	B*	A	Detection of maternal-specific allele	If A >B : fetal haplotype HapII<br>if B >A : fetal haplotype HapI	(1-f)/(2-f)	1/(2-f)
4X	R	A*B	B	AA*	A*	B	Detection of maternal-specific allele	If B >A : fetal haplotype HapII<br>if A >B : fetal haplotype HapI	(1-f)/(2-f)	1/(2-f)
WARNING_1	NA	AA	A	BB	NA	NA	Genotype Combination Impossible		NA	NA
WARNING_1	NA	AA	B	BB	NA	NA	Genotype Combination Impossible		NA	NA
WARNING_1	NA	BB	A	AA	NA	NA	Genotype Combination Impossible		NA	NA
WARNING_1	NA	BB	B	AA	NA	NA	Genotype Combination Impossible		NA	NA
WARNING_2	NA	AB	A	AB	NA	NA	Genotype Combination Non Informative		NA	NA
WARNING_2	NA	AB	B	AB	NA	NA	Genotype Combination Non Informative		NA	NA
WARNING_2	NA	AA	B	AA	NA	NA	Genotype Combination Non Informative		NA	NA
WARNING_2	NA	AA	B	AB	NA	NA	Genotype Combination Non Informative		NA	NA
WARNING_2	NA	BB	A	AB	NA	NA	Genotype Combination Non Informative		NA	NA
WARNING_2	NA	BB	A	BB	NA	NA	Genotype Combination Non Informative		NA	NA
**Clinical setting**		**"Male Fetus" AND "Proband = Maternal Affected Close Relative, Male"**								
2	C	AA	A	A	NA	NA	Sequencing error rate	Quality Control	NA	NA
2	D	BB	B	B	NA	NA	Sequencing error rate	Quality Control	NA	NA
4X	E	A*B	A	A*	A*	B	Detection of maternal-specific allele	If A >B : fetal haplotype HapI<br>if B >A : fetal haplotype HapII	(1-f)/(2-f)	1/(2-f)
4X	F	AB*	B	B	B*	A	Detection of maternal-specific allele	If B >A : fetal haplotype HapI<br>if A >B : fetal haplotype HapII	(1-f)/(2-f)	1/(2-f)
4X	G	A*B	B	A*	A*	B	Detection of maternal-specific allele	If B >A : fetal haplotype HapII<br>if A >B : fetal haplotype HapI	(1-f)/(2-f)	1/(2-f)
4X	H	AB*	A	B	B*	A	Detection of maternal-specific allele	If A >B : fetal haplotype HapII<br>if B >A : fetal haplotype HapI	(1-f)/(2-f)	1/(2-f)
WARNING_1	NA	AA	A	B	NA	NA	Genotype Combination Impossible		NA	NA
WARNING_1	NA	AA	B	B	NA	NA	Genotype Combination Impossible		NA	NA
WARNING_1	NA	BB	B	A	NA	NA	Genotype Combination Impossible		NA	NA
WARNING_1	NA	BB	A	A	NA	NA	Genotype Combination Impossible		NA	NA
WARNING_2	NA	AA	B	A	NA	NA	Genotype Combination Non Informative		NA	NA
WARNING_2	NA	BB	A	B	NA	NA	Genotype Combination Non Informative		NA	NA

SNP type	SNP Subtype	Maternal Genotype	Paternal Genotype	Proband Genotype	Maternal at-risk HapI	Maternal non-at-risk HapII	Information from genotype	Result interpretation	SPRT analysis q_0	q_1
**Clinical setting**		**"Female Fetus" AND "Proband = Maternal Carrier/Affected Close Relative, Female"**								
1	A	AA	B	AB	NA	NA	Detection of paternal contribution	Fetal Fraction	NA	NA
1	B	BB	A	AB	NA	NA	Detection of paternal contribution	Fetal Fraction	NA	NA
1	E	AA	B	AA	NA	NA	Detection of paternal contribution	Fetal Fraction	NA	NA
1	F	BB	A	BB	NA	NA	Detection of paternal contribution	Fetal Fraction	NA	NA
2	A	AA	A	AA	NA	NA	Sequencing error rate	Quality Control	NA	NA
2	B	BB	B	BB	NA	NA	Sequencing error rate	Quality Control	NA	NA
2	E	AA	A	AB	NA	NA	Sequencing error rate	Quality Control	NA	NA
2	F	BB	B	AB	NA	NA	Sequencing error rate	Quality Control	NA	NA
4X	AA	A*B	A	AA*	A*	B		If A >B : fetal haplotype HapI<br>if A = B : fetal haplotype HapII	0.5	(1+f)/2
4X	BB	AB*	B	BB*	B*	A	Detection of maternal-specific allele	If B >A : fetal haplotype HapI<br>if A = B : fetal haplotype HapII	0.5	(1+f)/2
4X	QQ	AB*	A	BB*	B*	A	Detection of maternal-specific allele	If A >B : fetal haplotype HapII<br>if A = B : fetal haplotype HapI	(1-f)/2	0.5
4X	RR	A*B	B	AA*	A*	B	Detection of maternal-specific allele	If B >A : fetal haplotype HapII<br>if A = B : fetal haplotype HapI	(1-f)/2	0.5
	NA	AA	A	BB	NA	NA	Genotype Combination Impossible		NA	NA
WARNING_1	NA	AA	B	BB	NA	NA	Genotype Combination Impossible		NA	NA
WARNING_1	NA	BB	A	AA	NA	NA	Genotype Combination Impossible		NA	NA
WARNING_1	NA	BB	B	AA	NA	NA	Genotype Combination Impossible		NA	NA
WARNING_2	NA	AB	A	AB	NA	NA	Genotype Combination Non Informative		NA	NA
WARNING_2	NA	AB	B	AB	NA	NA	Genotype Combination Non Informative		NA	NA
**Clinical setting**		**"Female Fetus" AND "Proband = Maternal Affected Close Relative, Male"**								
1	C	AA	B	A	NA	NA	Detection of paternal contribution	Fetal Fraction	NA	NA
1	D	BB	A	B	NA	NA	Detection of paternal contribution	Fetal Fraction	NA	NA
2	C	AA	A	A	NA	NA	Sequencing error rate	Quality Control	NA	NA
2	D	BB	B	B	NA	NA	Sequencing error rate	Quality Control	NA	NA
4X	EE	A*B	A	A*	A*	B	Detection of maternal-specific allele	If A >B : fetal haplotype HapI<br>if A = B : fetal haplotype HapII	0.5	(1+f)/2
4X	FF	AB*	B	B	B*	A	Detection of maternal-specific allele	If B >A : fetal haplotype HapI<br>if A = B : fetal haplotype HapII	0.5	(1+f)/2
4X	GG	A*B	B	A*	A*	B	Detection of maternal-specific allele	If B >A : fetal haplotype HapII<br>if A = B : fetal haplotype HapI	(1-f)/2	0.5
4X	HH	AB*	A	B	B*	A	Detection of maternal-specific allele	If A >B : fetal haplotype HapII<br>if A = B : fetal haplotype HapI	(1-f)/2	0.5
WARNING_1	NA	AA	A	B	NA	NA	Genotype Combination Impossible		NA	NA
WARNING_1	NA	AA	B	B	NA	NA	Genotype Combination Impossible		NA	NA
WARNING_1	NA	BB	B	A	NA	NA	Genotype Combination Impossible		NA	NA
WARNING_1	NA	BB	A	A	NA	NA	Genotype Combination Impossible		NA	NA
