# Supplementary material for: Non-invasive prenatal diagnosis of single gene disorders with enhanced relative haplotype dosage analysis for diagnostic implementation
Source: PLoS One. 2023 Apr 24;18(4):e0280976. doi: 10.1371/journal.pone.0280976 (PMC10124834; doi:10.1371/journal.pone.0280976)
Supplement: S3 Table — ADMI = autosomal dominant maternal inheritance; ADPI = autosomal dominant paternal inheritance; ARI = autosomal recessive inheritance; RXLI = X-linked inheritance; AC = affected child; UC = unaffected child; AR = affected close relative; UR = unaffected close relative; DP = depth of sequencing; #SNP4 = SNP4 count; #FWD and #REV = halotype block count in either orientation; #MUA, #MUB, #MUX: mean count of SNP4α, SNP4β or SNP4 (X-linked inheritance); CS: concordance score; BS: block score; Preg = pregnancy number; Inh = genetic inheritance mode; Prob = proband. (PDF) [file pone.0280976.s012.pdf]

Supplemental Data for

Non-Invasive Prenatal Diagnosis of Single Gene Disorders with enhanced Relative Haplotype Dosage Analysis for diagnosis implementation

Mathilde Pacault, Camille Verebi, Magali Champion, Lucie Orhant, Alexandre Perrier, Emmanuelle Girodon, France Leturcq, Dominique Vidaud, Claude Férec, Thierry Bienvenu, Romain Daveau, Juliette Nectoux

Supplementary Table S3

Table S3 : Patients' information and NIPD results. ADMI = autosomal dominant maternal inheritance ; ADPI = autosomal dominant paternal inheritance ; ARI = autosomal recessive inheritance ; RXLI = X-linked inheritance ; AC = affected child ; UC = unaffected child ; AR = affected close relative ; UR = unaffected close relative ; DP = depth of sequencing ; #SNP4 = SNP4 count ; #FWD and #REV = halotype block count in either orientation ; #MUA, #MUB, #MUX : mean count of SNP4α, SNP4β or SNP4 (X-linked inheritance) ; CS : concordance score ; BS : block score ; Preg = pregnancy number ; Inh = genetic inheritance mode ; Prob = proband.

Family	Preg	Maternal variant genomic position	Paternal variant genomic position	Inh mode	Proband's status	Gestational age	Target gene	Fetal fraction	cfDNA DP
2_1	1	7:117199646_117199648del	7:117199646_117199648del	ARI	AC	10 w + 4 d	*CFTR*	5.4	272.3
2_2	2	7:117199646_117199648del	7:117199646_117199648del	ARI	AC	10 w + 1 d	*CFTR*	12	168.3
2_2	3	7:117199646_117199648del	7:117199646_117199648del	ARI	AC	10 w + 2 d	*CFTR*	10.2	84.3
2_3	4	7:117199646_117199648del	7:117199646_117199648del	ADMI_AC ADPI_UC	AC	10 w + 1 d	CFTR	11.1	90.6
2_4	5	7:117199646_117199648del	7:117199646_117199648del	ARI	AC	9 w + 5 d	*CFTR*	8.2	87.6
2_5	6	7:117199646_117199648del	7:117199646_117199648del	ARI	AC	7 w + 0 d	*CFTR*	7.5	70.3
2_6	7	7:117199646_117199648del	7:117199646_117199648del	ARI	AC	9 w + 6 d	*CFTR*	14.6	55.1
2_7	8	7:117199522_117251704	7:117199522_117251704	ARI	AC	10 w + 0 d	*CFTR*	7.3	208.9
2_8	9	7:117199646_117199648del	7:117199646_117199648del	ARI	UC	10 w + 2 d	*CFTR*	9.6	62
2_8	10	7:117199646_117199648del	7:117199646_117199648del	ADMI_UC ADPI_AC	UC	10 w + 3 d	*CFTR*	12.9	187.8
2_9	11	7:117227792	7:117199646_117199648del	ARI	UC	10 w + 5 d	*CFTR*	12.1	65.6
2_10	12	7:117267722del	7:117305619	ARI	UC	15 w + 0 d	*CFTR*	6.7	183
2_11	13	7:117199645_117199647	7:117199645_117199647	ARI	AC	10 w + 6 d	*CFTR*	16.9	161.8
2_12	14	7:117149197	7:117280015	ARI	AC	10 w + 2 d	*CFTR*	4.5	62.3
2_13	15	7:117199646_117199648del	7:117199646_117199648del	ADMI_AC ADPI_UC	AC	16 w + 4 d	*CFTR*	10.3	49.2
2_13	16	7:117199646_117199648del	7:117199646_117199648del	ARI	AC	10 w + 1 d	*CFTR*	7.1	59.6
2_14	17	7:117199646_117199648del	7:117199646_117199648del	ARI	AC	9 w + 0 d	*CFTR*	5.9	115.7
2_15	18	7:117199646_117199648del	7:117199646_117199648del	ADMI_AC ADPI_UC	AC	8 w + 2 d	*CFTR*	13.2	39.9
2_15	19	7:117199646_117199648del	7:117199646_117199648del	ARI	AC	10 w + 1 d	*CFTR*	11.8	60.7
2_16	20	7:117199646_117199648del	7:117199646_117199648del	ADMI_AC ADPI_UC	AC	11 w + 0 d	*CFTR*	11.4	120.6
2_16	21	7:117199646_117199648del	7:117199646_117199648del	ARI	AC	9 w+ 4 d	*CFTR*	9.7	54.9
2_17	22	7:117227860	7:117292905	ARI	AC	12 w + 4 d	*CFTR*	17.1	265.4
2_18	23	7:117199646_117199648del	7:117199646_117199648del	ADMI_UC ADPI_AC	UC	11 w + 0 d	*CFTR*	8	62.7
2_18	24	7:117199646_117199648del	7:117199646_117199648del	ARI	AC	9 w + 4 d	*CFTR*	12.4	56.6
2_19	25	7:117199646_117199648del	7:117199646_117199648del	ARI	AC	12 w + 4 d	*CFTR*	15.5	54.5
2_20	26	17:29657391	NA	ADMI	UR	8 w + 1 d	*NF1*	8.6	55.7
2_21	27	17:29528504_29541468del	NA	ADMI	AC	10 w + 0 d	*NF1*	9.8	104.7
2_22	28	17:29677250_29677251del	NA	ADMI	AC	6 w + 2 d	*NF1*	8.3	79.1
2_23	29	17:29422388_29496908del	NA	ADMI	UC	12 w + 4 d	*NF1*	13.1	74.1
2_24	30	17:29667545	NA	ADMI	AC	10 w + 4 d	*NF1*	9.2	53.9
2_25	31	17:29661871_29661872del	NA	ADMI	AC	10 w + 0 d	*NF1*	10.9	205.9
2_26	32	17:29586142dup	NA	ADMI	AC	10 w + 4 d	*NF1*	6.8	73.5
2_26	32	17:29586142dup	NA	ADMI	AC	10 w + 4 d	/	6.9	79.3
2_26	33	17:29586142dup	NA	ADMI	AC	10 w + 0 d	*NF1*	8.1	66.3
2_26	33	17:29586142dup	NA	ADMI	AC	10 w + 0 d	/	7.9	70.7
2_26	34	17:29586142dup	NA	ADMI	AC	10 w + 4 d	*NF1*	6.9	95.9
2_26	34	17:29586142dup	NA	ADMI	AC	10 w + 4 d	/	6.9	95
2_27	35	17:29553492	NA	ADMI	UC	10 w + 0 d	*NF1*	9.5	121.3
2_27	36	17:29553492	NA	ADMI	AC	11 w + 3 d	*NF1*	7.4	105.3

Family	Preg	SNP3A total	SNP3A HapIII	SNP3A HapIV	SNP3B total	SNP3B HapIII	SNP3B HapIV	#SNP4	#FWD	#REV	#MUA	#MUB	CS	BS	NI maternal transmission	Fetal haplotype	NI paternal transmission	Fetal haplotype
2_1	1	386	0	351	99	0	0	813	40	41	27.3	13.7	0.97	0.8	HapII	HapII	HapIV	HapIV
2_2	2	436	0	1	139	132	0	406	83	84	4.9	4.4	0.99	0.97	HapI	HapI	HapIII	HapIII
2_2	3	637	0	0	173	140	0	566	34	33	17.5	12.2	0.96	0.79	HapI	HapI	HapIII	HapIII
2_3	4	481	0	396	471	0	0	464	34	33	14.1	13.6	0.99	0.76	HapI	HapI	HapIV	HapIV
2_4	5	99	0	5	342	165	0	371	12	12	33.5	25.8	0.91	0.55	HapI	HapI	HapIII	HapIII
2_5	6	354	0	286	565	2	0	396	19	20	17.4	28.1	0.93	0.62	HapII	HapII	HapIV	HapIV
2_6	7	82	0	69	270	0	0	598	55	55	13.3	10.3	0.98	0.85	HapI	HapI	HapIV	HapIV
2_7	8	558	0	0	?	?	?	249	36	36	6.9	0	0.99	0.93	HapI	HapI	HapIII	HapIII
2_8	9	230	0	0	94	85	0	541	35	35	16.6	14.9	0.96	0.77	HapII	HapII	HapIII	HapIII
2_8	10	245	0	231	107	0	0	619	117	117	5.7	5.1	0.99	1	HapII	HapII	HapIV	HapIV
2_9	11	392	0	0	291	246	0	436	41	40	11	10.6	0.97	0.8	HapI	HapI	HapIII	HapIII
2_10	12	279	0	0	348	316	0	654	46	42	16.1	13.8	0.79	0.82	HapII	HapII	HapIII	HapIII
2_11	13	309	0	0	174	163	0	594	164	167	3.6	3.6	0.99	1	HapI	HapI	HapIII	HapIII
2_12	14	422	0	110	61	1	0	512	6	6	68.8	118.5	0.8	0.37	NC	HapI	NC	HapIII
2_13	15	133	0	0	310	228	0	573	28	28	30	14.3	0.95	0.71	HapI	HapI	HapIII	HapIII
2_13	16	135	0	75	316	1	0	586	19	19	33.9	27.4	0.91	0.64	HapI	HapI	HapIV	HapIV
2_14	17	201	0	2	624	534	0	342	11	11	32	30.8	0.8	0.52	NC	HapI	HapIII	HapIII
2_15	18	548	0	5	199	163	0	517	27	27	20.6	17	0.98	0.72	HapI	HapI	HapIII	HapIII
2_15	19	572	0	484	225	0	0	571	43	43	16.4	9.5	0.93	0.82	HapI	HapI	HapIV	HapIV
2_16	20	561	0	0	344	300	0	567	60	62	8.5	10	0.97	0.89	HapI	HapI	HapIII	HapIII
2_16	21	455	0	351	238	0	0	498	22	21	20	26.8	0.91	0.66	HapI	HapI	HapIV	HapIV
2_17	22	365	0	0	296	274	0	535	230	232	2.3	2.3	1	1	HapII	HapII	HapIII	HapIII
2_18	23	617	0	2	?	?	?	244	13	13	18.8	0	0.95	0.71	HapI	HapI	HapIII	HapIII
2_18	24	624	0	1	?	?	?	234	25	26	9.2	0	0.98	0.85	HapII	HapII	HapIII	HapIII
2_19	25	216	0	168	284	0	0	477	42	42	13.3	7.5	0.98	0.83	HapI	HapI	HapIV	HapIV
2_20	26	*NA*	*NA*	*NA*	*NA*	*NA*	*NA*	357	24	24	12	15.6	0.95	0.71	HapII	HapII	*NA*	*NA*
2_21	27	*NA*	*NA*	*NA*	*NA*	*NA*	*NA*	596	46	46	15.6	11.2	0.97	0.82	HapII	HapII	*NA*	*NA*
2_22	28	*NA*	*NA*	*NA*	*NA*	*NA*	*NA*	638	33	32	19	21.5	0.97	0.75	HapI	HapI	*NA*	*NA*
2_23	29	*NA*	*NA*	*NA*	*NA*	*NA*	*NA*	557	70	69	8.3	7.5	0.98	0.92	HapII	HapII	*NA*	*NA*
2_24	30	*NA*	*NA*	*NA*	*NA*	*NA*	*NA*	431	12	14	40.5	29.6	0.84	0.54	HapI	HapI	*NA*	*NA*
2_25	31	*NA*	*NA*	*NA*	*NA*	*NA*	*NA*	472	91	91	5.2	5.2	0.99	0.98	HapI	HapI	*NA*	*NA*
2_26	32	*NA*	*NA*	*NA*	*NA*	*NA*	*NA*	459	17	17	24.7	32.1	0.59	0.6	HapI	HapI	*NA*	*NA*
2_26	32	*NA*	*NA*	*NA*	*NA*	*NA*	*NA*	439	15	15	28.4	31	0.86	0.58	HapI	HapI	*NA*	*NA*
2_26	33	*NA*	*NA*	*NA*	*NA*	*NA*	*NA*	452	20	18	26.2	20.9	0.84	0.64	HapI	HapI	*NA*	*NA*
2_26	33	*NA*	*NA*	*NA*	*NA*	*NA*	*NA*	427	19	16	24.9	24	0.61	0.62	HapI	HapI	*NA*	*NA*
2_26	34	*NA*	*NA*	*NA*	*NA*	*NA*	*NA*	436	29	30	15.7	13.4	0.71	0.74	HapI	HapI	*NA*	*NA*
2_26	34	*NA*	*NA*	*NA*	*NA*	*NA*	*NA*	432	30	30	15.1	13.3	0.67	0.74	HapI	HapI	*NA*	*NA*
2_27	35	*NA*	*NA*	*NA*	*NA*	*NA*	*NA*	485	46	45	11.8	8.5	0.97	0.84	HapI	HapI	*NA*	*NA*
2_27	36	*NA*	*NA*	*NA*	*NA*	*NA*	*NA*	492	28	26	20.4	14	0.96	0.73	HapII	HapII	*NA*	*NA*

Family	Preg	Maternal variant genomic position	Inh mode	Fetal gender	Prob gender	Prob status	Gestational age	Target gene	Fetal fraction	cfDNA DP	#SNP4	#FWD	#REV	#MUX	CS	BS	Non-invasive transmission	Fetal haplotype
2_28	37	X:31893385_32235089del	RXLI	XY	XY	AR	10 w + 4 d	*DMD*	6.8	107.2	1699	64	65	26.3	0.98	0.9	HapII	HapII
2_29	38	X:31525571_31838091del	RXLI	XY	XY	UC	13 w + 4 d	*DMD*	5.6	51.8	1556	19	18	84.2	0.84	0.63	HapII	HapII
2_30	39	X:31792196_32235089del	RXLI	XY	XY	AR	11 w + 2 d	*DMD*	10.2	101.8	2273	152	155	14.8	0.89	1	HapII	HapII
2_31	40	X:33038290_33357493del	RXLI	XY	XY	UC	9 w + 3 d	*DMD*	19.3	101.2	1657	187	187	8.9	0.71	1	HapI	HapI
2_31	41	X:33038291_33357494	RXLI	XY	XY	AC	12 w + 4 d	*DMD*	18.7	233.3	1226	330	336	3.7	0.67	1	HapI	HapI
2_32	42	X:32583818	RXLI	XY	XY	AC	10 w + 0 d	*DMD*	9.3	147.5	2052	166	162	12.5	0.93	1	HapI	HapI
2_33	43	X:31854936_31986456del	RXLI	XY	XY	AC	10 w + 0 d	*DMD*	3.6	106.9	1864	17	17	109.6	0.91	0.62	HapII	HapII
2_34	44	X:31697577_31792196del	RXLI	XY	XY	UC	14 w + 2 d	*DMD*	4.4	111.9	1761	46	46	38.3	1	0.83	HapI	HapI
2_35	45	X:31191698_31222106dup	RXLI	XY	XX	AR	9 w + 6 d	*DMD*	9.4	87	1085	45	44	24.4	0.99	0.82	HapII	HapII
2_36	46	X:32360279	RXLI	XY	XY	AC	10 w + 4 d	*DMD*	9.4	73.2	1469	47	48	30.9	0.99	0.84	HapII	HapII
2_37	47	X:31191655_31198598	RXLI	XY	XY	AR	10 w + 5 d	*DMD*	11.4	170.7	1221	166	166	7.4	1	1	HapII	HapII
2_38	48	X:31838079_32235090	RXLI	XY	XY	AR	11 w + 0 d	*DMD*	7.4	246.9	1268	170	172	7.4	0.99	1	HapI	HapI
2_39	49	X:32305819_32632419	RXLI	XY	XX	AR	10 w + 1 d	*DMD*	4.5	232.7	621	25	25	24.8	0.6	0.7	NC	HapI
2_40	50	X:31792196_31893385dup	RXLI	XY	XY	AC	10 w + 3 d	*DMD*	2.9	103.1	2169	15	15	144.6	0.97	0.59	HapI	HapI
2_41	51	X:31645969	RXLI	XY	XY	AR	12 w + 2 d	*DMD*	7.1	192.7	1350	91	92	14.8	0.53	0.98	HapI	HapI
2_42	52	X:32383136_32404582	RXLI	XY	XY	AR	15 w + 4 d	*DMD*	4.8	211.3	1270	59	59	21.5	1	0.89	HapI	HapI
2_43	53	X:32482745_32827701del	RXLI	XY	XX	AR	10 w + 3 d	*DMD*	6.6	105	786	26	26	30.2	0.95	0.71	HapI	HapI
2_43	54	X:32482745_32827701del	RXLI	XY	XX	AR	11 w + 6 d	*DMD*	8.3	83	770	27	27	28.5	0.98	0.72	HapI	HapI
2_44	55	X:31838079_31947816	RXLI	XY	XY	AR	10 w + 3 d	*DMD*	11	249.9	1082	223	224	4.8	0.81	1	HapII	HapII
2_45	56	X:32407617_32459431	RXLI	XY	XY	AR	11 w + 0 d	*DMD*	6.9	209.7	1219	69	67	17.9	0.52	0.92	HapI	HapI
