# Supplementary material for: Non-invasive prenatal diagnosis of single gene disorders with enhanced relative haplotype dosage analysis for diagnostic implementation
Source: PLoS One. 2023 Apr 24;18(4):e0280976. doi: 10.1371/journal.pone.0280976 (PMC10124834; doi:10.1371/journal.pone.0280976)
Supplement: S4 Table — The p-values are adjusted for multiple comparisons. (PDF) [file pone.0280976.s013.pdf]

Supplemental Data for

Non-Invasive Prenatal Diagnosis of Single Gene Disorders with enhanced Relative Haplotype Dosage Analysis for diagnosis implementation

Mathilde Pacault, Camille Verebi, Magali Champion, Lucie Orhant, Alexandre Perrier, Emmanuelle Girodon, France Leturcq, Dominique Vidaud, Claude Férec, Thierry Bienvenu, Romain Daveau, Juliette Nectoux

Supplementary Table S4

Table S4 : p-values obtained after testing differences between extensive/restrictive discrimination of heterozygous position in gDNA for both scores using Wilcoxon tests. The p-values are adjusted for multiple comparisons.

	Block score	Concordance score
extensive vs. restrictive	4.8e^{-20}	1.1e^{-8}
