# Supplementary material for: Non-invasive prenatal diagnosis of single gene disorders with enhanced relative haplotype dosage analysis for diagnostic implementation
Source: PLoS One. 2023 Apr 24;18(4):e0280976. doi: 10.1371/journal.pone.0280976 (PMC10124834; doi:10.1371/journal.pone.0280976)
Supplement: S5 Table — The global p-value (Friedman test) is indicated in brackets on the top of the table. All p-values are adjusted for multiple comparisons. (PDF) [file pone.0280976.s014.pdf]

Supplemental Data for

Non-Invasive Prenatal Diagnosis of Single Gene Disorders with enhanced Relative Haplotype Dosage Analysis for diagnosis implementation

Mathilde Pacault, Camille Verebi, Magali Champion, Lucie Orhant, Alexandre Perrier, Emmanuelle Girodon, France Leturcq, Dominique Vidaud, Claude Férec, Thierry Bienvenu, Romain Daveau, Juliette Nectoux

Supplementary Table S5

Table S5 : p-values obtained after testing differences between each pair of variables PDP for each value of NDP using Wilcoxon tests. The global p-value (Friedman test) is indicated in brackets on the top of the table. All p-values are adjusted for multiple comparisons.

		Block score ($4.8e^{-24}$)				Concordance score ($7.3e^{-1}$)			
NDP	PDP	15	30	45	60	15	30	45	60
8	15	•	1.4e^{-1}	2.6e^{-4}	2.5e^{-11}	•	1	1	1
	30		•	1.1e^{-2}	3.1e^{-10}		•	1	1
	45			•	2.7e^{-10}			•	1
	60				•				•
15	15	•	1.4e^{-1}	2.6e^{-4}	2.5e^{-11}	•	1	1	1
	30		•	1.1e^{-2}	3.1e^{-10}		•	1	1
	45			•	2.7e^{-10}			•	1
	60				•				•
30	15	•	1.4e^{-1}	2.6e^{-4}	2.5e^{-11}	•	1	1	1
	30		•	1.1e^{-2}	3.1e^{-10}		•	1	1
	45			•	2.7e^{-10}			•	1
	60				•				•
