# Supplementary material for: Non-invasive prenatal diagnosis of single gene disorders with enhanced relative haplotype dosage analysis for diagnostic implementation
Source: PLoS One. 2023 Apr 24;18(4):e0280976. doi: 10.1371/journal.pone.0280976 (PMC10124834; doi:10.1371/journal.pone.0280976)
Supplement: S7 Table — p-values for ANOVA tests are indicated in brackets. (PDF) [file pone.0280976.s016.pdf]

Supplemental Data for

Non-Invasive Prenatal Diagnosis of Single Gene Disorders with enhanced Relative Haplotype Dosage Analysis for diagnosis implementation

Mathilde Pacault, Camille Verebi, Magali Champion, Lucie Orhant, Alexandre Perrier, Emmanuelle Girodon, France Leturcq, Dominique Vidaud, Claude Férec, Thierry Bienvenu, Romain Daveau, Juliette Nectoux

Supplementary Table S7

Table S7 : Sobol indices indicating the effect of all parameters (fetal fraction, number of SNPs and sequencing depth) and their combinations on the results quality ($I_{\mathcal{S}_b}$ and $I_{\mathcal{S}_c}$ for block and concordance scores respectively). p-values for ANOVA tests are indicated in brackets.

Parameters	$I_{\mathcal{S}_b}$ (p-value)	$I_{\mathcal{S}_c}$ (p-value)
Fetal fraction	57.9 ($< e^{-16}$)	41.0 ($< e^{-16}$)
Number of SNPs	13.6 ($< e^{-16}$)	5.5 ($< e^{-16}$)
Sequencing depth	8.4 ($< e^{-16}$)	4.1 ($< e^{-16}$)
Fetal fraction, number of SNPs	$3.7e^{-1}$ ($< e^{-16}$)	4.2 ($< e^{-16}$)
Fetal fraction, sequencing depth	2.2e^{-1} ($< e^{-16}$)	2.8 ($< e^{-16}$)
Number of SNPs, sequencing depth	3.2e^{-1} ($< e^{-16}$)	5.0e^{-1} ($< e^{-16}$)
Fetal fraction, number of SNPs, sequencing depth	7.0e^{-1} ($< e^{-16}$)	1.6e^{-2} ($9.1e^{-2}$)
