# Supplementary material for: Non-invasive prenatal diagnosis of single gene disorders with enhanced relative haplotype dosage analysis for diagnostic implementation
Source: PLoS One. 2023 Apr 24;18(4):e0280976. doi: 10.1371/journal.pone.0280976 (PMC10124834; doi:10.1371/journal.pone.0280976)
Supplement: S1 File — (PDF) [file pone.0280976.s017.pdf]

Supplemental Data for

Non-Invasive Prenatal Diagnosis of Single Gene Disorders with enhanced Relative Haplotype Dosage Analysis for diagnosis implementation

Mathilde Pacault, Camille Verebi, Magali Champion, Lucie Orhant, Alexandre Perrier, Emmanuelle Girodon, France Leturcq, Dominique Vidaud, Claude Férec, Thierry Bienvenu, Romain Daveau, Juliette Nectoux

Supplementary Methods

Patients recruitment and sample processing. Written informed consent was obtained prior to venipuncture. Inclusion criteria were an ongoing pregnancy for a couple at risk of 25% or 50% of transmitting one of the following SGD : Cystic Fibrosis (CF), Neurofibromatosis type I (NF1), Duchenne Muscular Dystrophy (DMD), Hemophilia (HM), regardless of the parental mutations. Exclusion criteria were the absence of conventional PND, as fetal DNA is needed in the following steps of the analysis for development purposes. Because of too few SNPs captured around the *F8* gene, HM families were not included in our test cohort.

Plasma sample collection and processing was done as previously described (1). cfDNA was extracted from 10 mL of plasma using the QIAmp Circulating Nucleic Acid (Qiagen, Valencia, CA, USA) following manufacturer's instructions. The extracted cfDNA was eluted in 100 μ L of elution buffer and stored at 20 ° C until use. cfDNA was not quantified after extraction.

Parental and fetal gDNA obtained after chorionic villus sampling or amniotic fluid ponction were retrieved once the PND result had been transmitted. When available, the proband's gDNA was also retrieved.

Biotinylated probes (Kapa Biosystems, Roche Sequencing, MA, USA) were designed to target the coding regions and frequently mutated non-coding regions of our genes of interest, namely *CFTR*, *NF1*, *DMD*, *F8*, *F9*, as well as biallelic SNPs with a minor allele frequency higher than 20% in a 2-Mb flanking region upstream and downstream of each gene. To increase precision in sequencing quality and fetal fraction assessment, coding regions of *ZFY* and *ZFX* as well as other biallelic SNPs with a minor allele frequency higher than 20% distributed all along the genome were added to the target region.

For each family, parental and fetal gDNA, cfDNA and proband gDNA when available were sequenced in parallel. DNA libraries were prepared using the Kapa HyperPlus Library Preparation Kit and the HyperCap Target Enrichment kit or KAPA HyperCapture kit (Kapa Biosystems, Roche Sequencing, MA, USA) from 100ng gDNA or 60 µL cfDNA, following manufacturer's instructions, with minor adaptations for cfDNA. Briefly, the fragmentation step was not performed, as it is already fragmented (2, 3). Adapters ligation reaction was incubated overnight, at a lower temperature (16°C) for the HyperCapture protocole, to maximise efficiency. Finally, because of the lower quantity of the starting material, 9 amplification cycles were done during pre-capture PCR for cfDNA, whereas 6 cycles were done for gDNA.

A maximum of 12 samples were pooled before hybridization, following manufacturer's recommendations. In order to increase sequencing depth, cfDNA libraries were sequenced in duplicates.

Amplified DNA and captured libraries were quantified using the Qubit HS dsDNA assay (Therm Fisher Scientific, MA, USA) and qualified using the 2100 Bioanalyzer system (Agilent, Ca, USA). After pooling of every hybridization reaction, the final library was loaded at a concentration of 2 pM with 0.1% PhiX control and sequenced using 75-cycle paired-end reads with NextSeq Mid Output 150-cycle v2 reagents on an Illumina NextSeq500.

Sequencing data analysis. After sequencing, bam files are generated for each sample from fastq files. For each family, a unique pileup is generated, counting the number of read for each base, from parental at-risk- and non-at-risk haplotypes, at every genomic position targeted in our capture panel.

After sequencing, FASTQ files were trimmed with BBDuk (Joint Genome Institute), mapped using BWA-MEM ((4)) and post-processed through a standard GATK local re-alignment and BQSR procedure (5). In order to retain information at loci that would be homozygous for the reference allele, we chose to generate an analysis file for each family (parents, proband, fetal DNA, cfDNA) using a pileup rather than a regular variant calling approach, from which we extracted the number of read for each nucleotide at every genomic position targeted in our capture panel.

SNP typing and haplotypes quantification was performed with a dedicated Perl script (Programming perl. Wall, Larry and Christiansen, Tom and Orwant, Jon. O'Reilly Media, Inc. 2000). Thresholds to call homozygous or heterozygous genotypes were fixed to higher than 0.85 or lower than 0.15, and between 0.35 and 0.65 allelic ratio, respectively.

SNP from each category are then extracted from these pileups for NIPD analysis, which is conducted with R (R: A language and environment for statistical computing. R Foundation for Statistical Computing, Vienna, Austria. URL https://www.R-project.org) in four steps, namely sequencing error rate, fetal fraction, qualitative detection of paternal transmission and quantitative detection by RHDO of maternal transmission.

Sequencing error rate. Sequencing error rate density for each family was estimated using SNP2, corresponding to positions where both parents are homozygous for the same allele.

Estimating the fetal fraction. Fetal fraction was estimated without prior knowledge of the SNP category, using the minor allele frequency distribution (MAF). In this density plot, the first peak is likely to correspond to the frequency of the fetal-specific allele at positions where the fetus is heterozygous in a homozygous maternal background, allowing to infer the fetal fraction from twice this haploid frequency. Estimation using SNP1 distribution, where both parents are homozygous for different alleles, was retained for comparison purposes. The fractional fetal DNA concentration is evaluated using the formula $f = 2p/(p+q)$ where p is the count of sequenced reads from the fetal-specific allele and q is the count of sequenced reads from the maternal and fetal shared allele.

Detecting paternal transmission. Paternal haplotypes were named HapIII and HapIV for the mutant-linked haplotype and the wild-type-linked haplotype, respectively. Paternal inheritance was qualitatively determined using SNP3, corresponding to positions where the father is heterozygous and the mother homozygous, as previously described (2), reported in Supplementary Tables **??** and **??**. SNP3 were divided in subcategories, depending on whether the paternal-specific allele was on HapIII or

HapIV. In other words, qualitative detection of a SNP3A/D/E/H/K/L SNP would be interpeted as "HapIV inherited", while qualitative detection of a ¨SNP3B/C/F/G/I/J SNP would be interpeted as "HapIII inherited". To take into account the sequencing error rate, only SNPs with an allelic frequency higher than three times the sequencing error rate were included for paternal inheritance analysis. We then introduced a minimal threshold for considering the paternal-specific allele as significantly detected called "min", corresponding to half the fetal fraction minus its standard deviation, representing the a lower estimate of the haploid fetal paternally-transmitted genome.

Detecting maternal transmission. Maternal haplotypes were named HapI and HapII for the mutant-linked haplotype and the wild-type-linked haplotype, respectively. RHDO was performed for SNP4, corresponding to positions where the mother is heterozygous and the father homozygous, followed by a Sequential Probability Ratio Test (SPRT)(6, 7), as previously described (2, 8, 9).

Briefly, the SPRT statistically detects an significant imbalance between alleles in maternal plasma by comparing the observed wild-type to mutant allele ratio with the expected one in both conditions (HapI or HapII fetal inheritance). A classification rule, which changes with the number of SNPs as the genome is explored, is defined in such a way that wrong decisions, also known as type I (probability of claiming HapII maternal inheritance instead of HapI) and type II (probability of claiming HapI maternal inheritance instead of HapII) errors, denoted by α and β, are controlled. Whenever the classification threshold is reached, fetal inheritance is established with an associated risk of missclassifications of α for HapII inheritance and β for HapI inheritance.

In eRHDO analysis for autosomal disorders, SNP4 are categorized into SNP4α, were the mutation-linked maternal allele is identical to the paternal allele, and SNP4β, were the mutation-linked maternal allele is different than the paternal allele (Supplementary Tables **??** and **??**). Concerning eRHDO analysis for X-linked disorders, no α or β categorization was done, as the paternal haplotype is not considered for fetal inheritance. However, distinction was made between the theoretical situations of male or female fetus, as SPRT bounds will vary. Four statistical analyses were performed separately, in forward and in reverse direction, and for SNP4α and SNP4β.

Simulated data. To evaluate the impact of biological and statistical parameters, namely the fetal fraction f, the number of tested SNP4 N, the sequencing depth and the statistical risks, we simulated samples of sequencing data by:

1. adding a uniform noise to the parameters fetal fraction and number of SNPs to increase the randomness of each sample,
2. generating a random number of SNP4α and SNP4β according to a a binomial distribution $\mathcal{B}(N_i, 1/2)$, with N_i the number of SNP4 corresponding to sample i,
3. generating the total number of reads of the fetus according to a Poisson-law with parameter corresponding to the sequencing depth,
4. simulating the fetal inheritance using the expected distribution of the wild-type allele ratio, as defined in the SPRT test, which depends on the situation (HapI or HapII fetus inheritance).

For more robustness, the experiments were replicated 100 times each in both conditions and for different parameters range values: f varying from 0.01 to 0.2, N from 0 to 2000, the sequencing depth from 30 to 300 and the statistical risks from 1/10000 to 5/100.

References

1. Gruber A, Pacault M, El Khattabi LA, Vaucouleur N, Orhant L, Bienvenu T, et al. Non-invasive prenatal diagnosis of paternally inherited disorders from maternal plasma: detection of NF1 and CFTR mutations using droplet digital PCR. Clin Chem Lab Med. 2018;56(5):728–738. doi:10.1515/cclm-2017-0689.
2. Lo YMD, Chan KCA, Sun H, Chen EZ, Jiang P, Lun FMF, et al. Maternal plasma DNA sequencing reveals the genome-wide genetic and mutational profile of the fetus. Sci Transl Med. 2010;2(61):61ra91. doi:10.1126/scitranslmed.3001720.
3. Chan KCA, Jiang P, Sun K, Cheng YKY, Tong YK, Cheng SH, et al. Second generation noninvasive fetal genome analysis reveals de novo mutations, single-base parental inheritance, and preferred DNA ends. Proc Natl Acad Sci U S A. 2016;113(50):E8159–E8168. doi:10.1073/pnas.1615800113.
4. Li H, Durbin R. Fast and accurate short read alignment with Burrows-Wheeler transform. Bioinformatics. 2009;25(14):1754–1760. doi:10.1093/bioinformatics/btp324.
5. McKenna A, Hanna M, Banks E, Sivachenko A, Cibulskis K, Kernytsky A, et al. The Genome Analysis Toolkit: a MapReduce framework for analyzing next-generation DNA sequencing data. Genome Res. 2010;20(9):1297–1303. doi:10.1101/gr.107524.110.
6. Wald A. Sequential Tests of Statistical Hypotheses. Ann Math Stat. 1945;16(2):117–186.
7. El Karoui N, Zhou W, Whittemore AS. Getting more from digital SNP data. Stat Med. 2006;25(18):3124–3133. doi:10.1002/sim.2379.
8. Parks M, Court S, Cleary S, Clokie S, Hewitt J, Williams D, et al. Non-invasive prenatal diagnosis of Duchenne and Becker muscular dystrophies by relative haplotype dosage. Prenat Diagn. 2016;36(4):312–320. doi:10.1002/pd.4781.
9. Chandler NJ, Ahlfors H, Drury S, Mellis R, Hill M, McKay FJ, et al. Noninvasive Prenatal Diagnosis for Cystic Fibrosis: Implementation, Uptake, Outcome, and Implications. Clin Chem. 2020;66(1):207–216. doi:10.1373/clinchem.2019.305011.
